# Crystal structure and supra­molecular features of a host–guest inclusion complex based on A1/A2-hetero-difunctionalized pillar[5]arene

**DOI:** 10.1107/S2056989024009216

**Published:** 2024-09-24

**Authors:** Mickey Vinodh, Talal F. Al-Azemi

**Affiliations:** a Department of Chemistry, Kuwait University, PO Box 5969, Safat 13060, Kuwait; Universidad Nacional Autónoma de México, México

**Keywords:** crystal structure, hetero difunctionalized pillar[5]arene, inclusion complex, supra­molecular dimer

## Abstract

A host–guest supra­molecular inclusion complex was obtained from the co-crystallization of A1/A2-bromo­but­oxy-hy­droxy difunctionalized pillar[5]arene (**PilButBrOH**) with adipo­nitrile (**ADN**), C_47_H_53.18_Br_0.82_O_10_·C_6_H_8_N_2_. The adipo­nitrile guest is stabilized within the electron-rich cavity of the pillar[5]arene host *via* multiple C—H⋯O and C—H⋯π inter­actions. Both functional groups on the macrocyclic rim are engaged in supra­molecular inter­actions with an adjacent inclusion complex *via* hydrogen-bonding (O—H⋯N or C—H⋯Br) inter­actions, resulting in the formation of a supra­molecular dimer in the crystal structure.

## Abstract 2

The crystal structure of an A1/A2-bromo­but­oxy-hy­droxy difunctionalized pillar[5]arene that encapsulates an adipo­nitrile mol­ecule in its cavity is reported and the supra­molecular features of this inclusion complex are discussed.

## 1. Chemical context

Pillar[*n*]arenes are highly studied scaffolds among the macrocyclic compounds because of their ease of formation, rigid shape, capacious cavities, and well-defined conformations (Ogoshi *et al.*, 2016 ▸). The ease of facile functionalization at the macrocyclic rims along with their adaptable capacity to create inclusion complexes with target guests (charged/neutral) *via* non-bonding inter­actions make pillararene systems inter­esting functional materials in supra­molecular chemistry (Guo *et al.*, 2018 ▸; Al-Azemi & Vinodh, 2020 ▸; Vinodh *et al.*, 2023 ▸; Yang *et al.*, 2024 ▸). Such tunable functionalilization and binding properties enables the pillararene family to find promising applications over multiple fields including drug delivery, nanomaterials, sensors, transmembrance channels, and catal­ysis (Guo *et al.*, 2020 ▸; Li *et al.*, 2020 ▸; Zhu *et al.*, 2021 ▸; Wang *et al.*, 2022*a* ▸; Zyryanov *et al.*, 2023 ▸). Selective manipulation of supra­molecular materials based on the pillararene framework could be achieved by carefully tuning these macrocycles with suitable functional groups. The design and synthesis of a numerous variety of mono-/di-/per-functionalized pillararenes and their supra­molecular inter­actions have been reported (Ogoshi *et al.*, 2016 ▸; Fang *et al.*, 2020 ▸). However, the incorporation of different kinds of functional groups on the same pillararene macrocycle has rarely been encountered even if such heterofunctional macrocycles are expected to be inter­esting supra­molecular systems (Al-Azemi & Vinodh, 2021 ▸, 2022 ▸). Previously, we reported the synthesis of macrocyclic systems comprising A1/A2 bromo­alk­oxy-hy­droxy pillar[5]arenes accompanied by their supra­molecular self assembly in solution (Al-Azemi & Vinodh, 2021 ▸). In this communication, we report the X-ray single crystal data of an inclusion complex comprising an A1/A2 bromo­but­oxy-hy­droxy difunctionalized pillar[5]arene host and an adipo­nitrile guest. The structural details of this pillar[5]arene system along with the supra­molecular inter­actions of this inclusion complex in its crystal network are addressed and discussed.
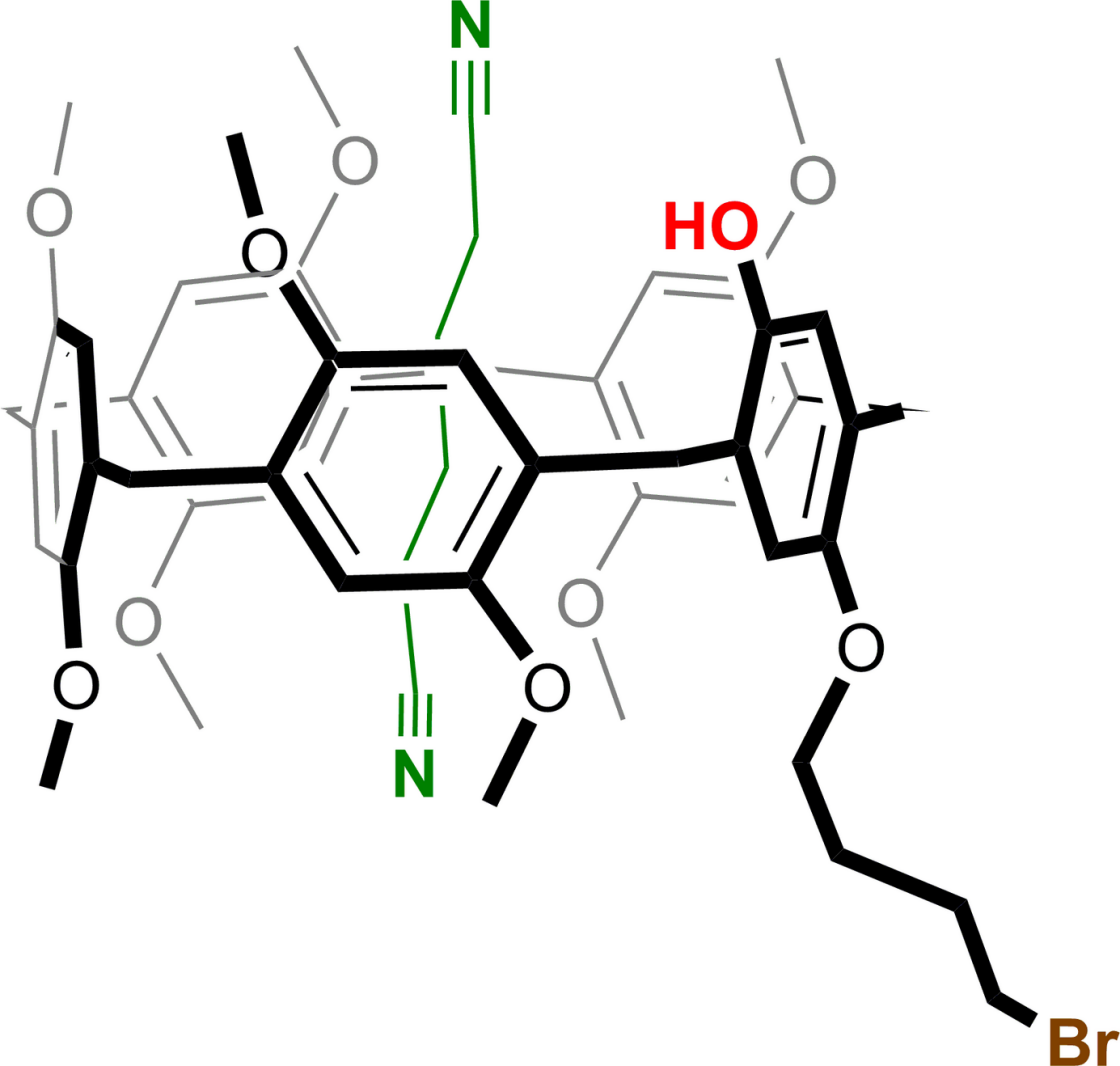


## 2. Structural commentary

**Figure 1:**
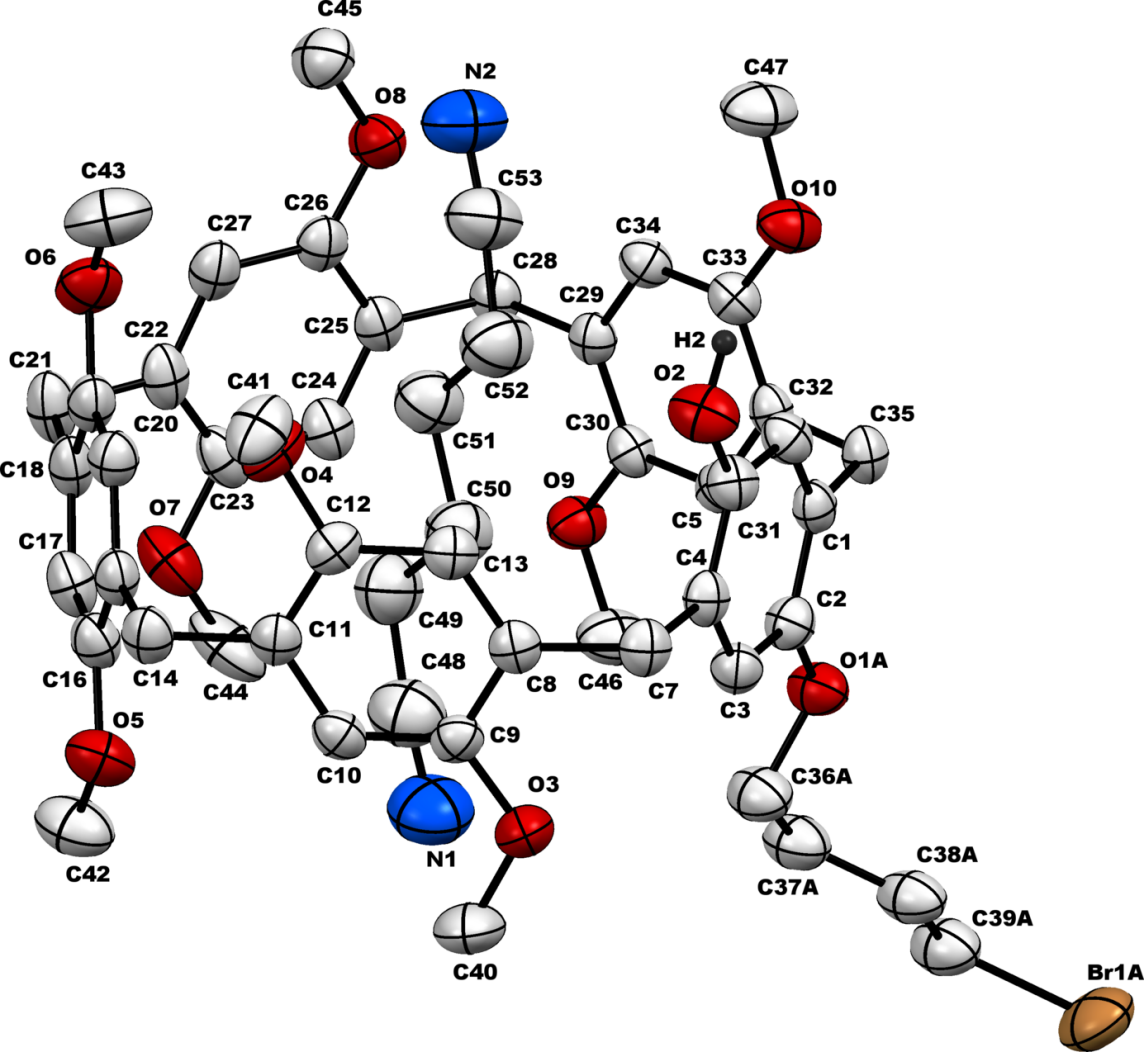
Crystal structure (displacement ellipsoid representation; 30% probability) of **PilButBrOH·ADN**. Only the major components of the disordered moieties are shown. Hydrogen atoms except the OH hydrogen are omitted for clarity.

**Figure 2:**
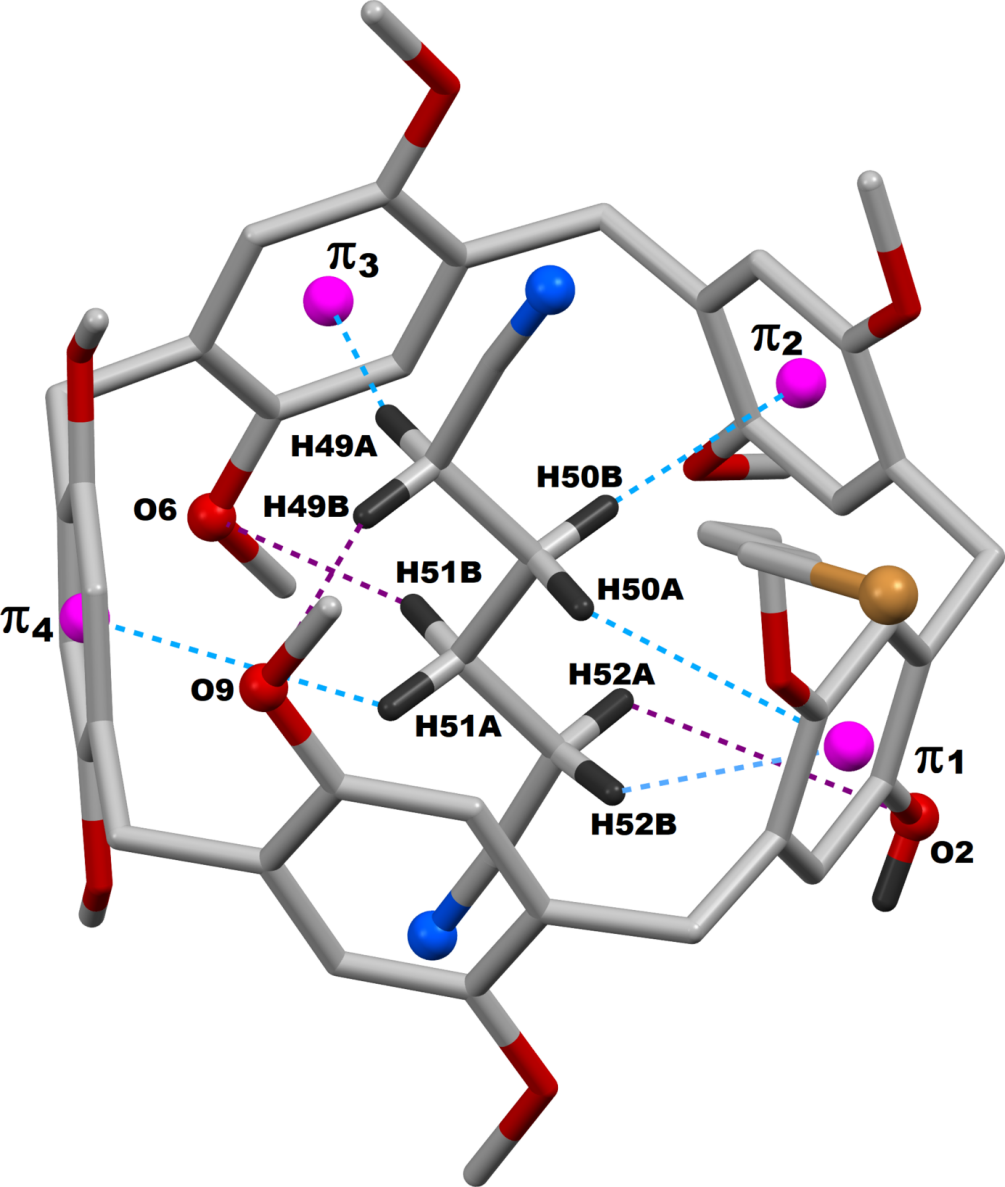
Inter­molecular inter­actions between the pillar[5]arene host and adipo­nitrile guest. π1–π4 are the centroids of the C1–C6, C8–C13, C15–C20 and C22–C27 phenyl rings, respectively.

The bromo­but­oxy-hy­droxy difunctionalized pillar[5]arene (**PilButBrOH**) crystallizes in the monoclinic crystal system, space group *P*2_1_/*n*. In the crystal structure, one mol­ecule of adipo­nitrile (**ADN**) is encapsulated within the cavity of the pillar[5]arene, resulting in the formation of a host–guest supra­molecular inclusion complex (**PilButBrOH·ADN)**. The structure of the pillar[5]arene is a penta­gonal-shaped macrocycle having *n*-bromo­but­oxy substitution, which is projected outward from the rim as depicted in Fig. 1 ▸. The hy­droxy group is oriented opposite to the *n*-bromo­but­oxy moiety and both these functional groups serve as active sites for supra­molecular inter­actions. The encapsulated guest adipo­nitrile mol­ecule engaged in multiple non-bonding inter­actions with its macrocyclic host *via* C—H⋯O or C—H⋯π inter­actions as shown in Fig. 2 ▸ and Table 1 ▸.

## 3. Supra­molecular features

**Figure 3:**
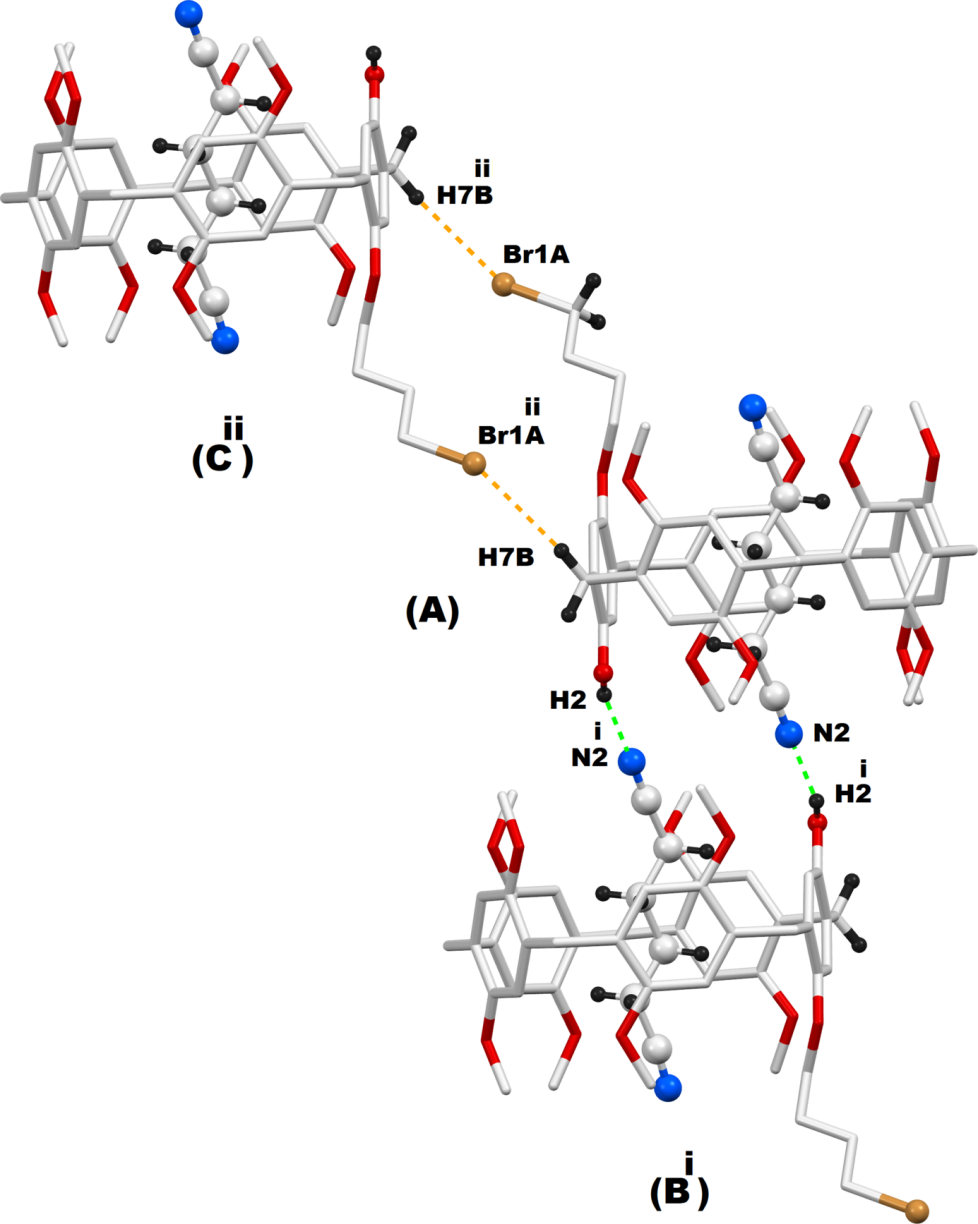
Pillar[5]arene–pillar[5]arene supra­molecular systems resulting from the hy­droxy as well as bromo­but­oxy-mediated dimeric inter­actions in **PilButBrOH·ADN**; Symmetry codes: (i) 1 − *x*, 1 − *y*, 2 − *z*; (ii) 1 − *x*, −*y*, 2 − *z*.

**Figure 4:**
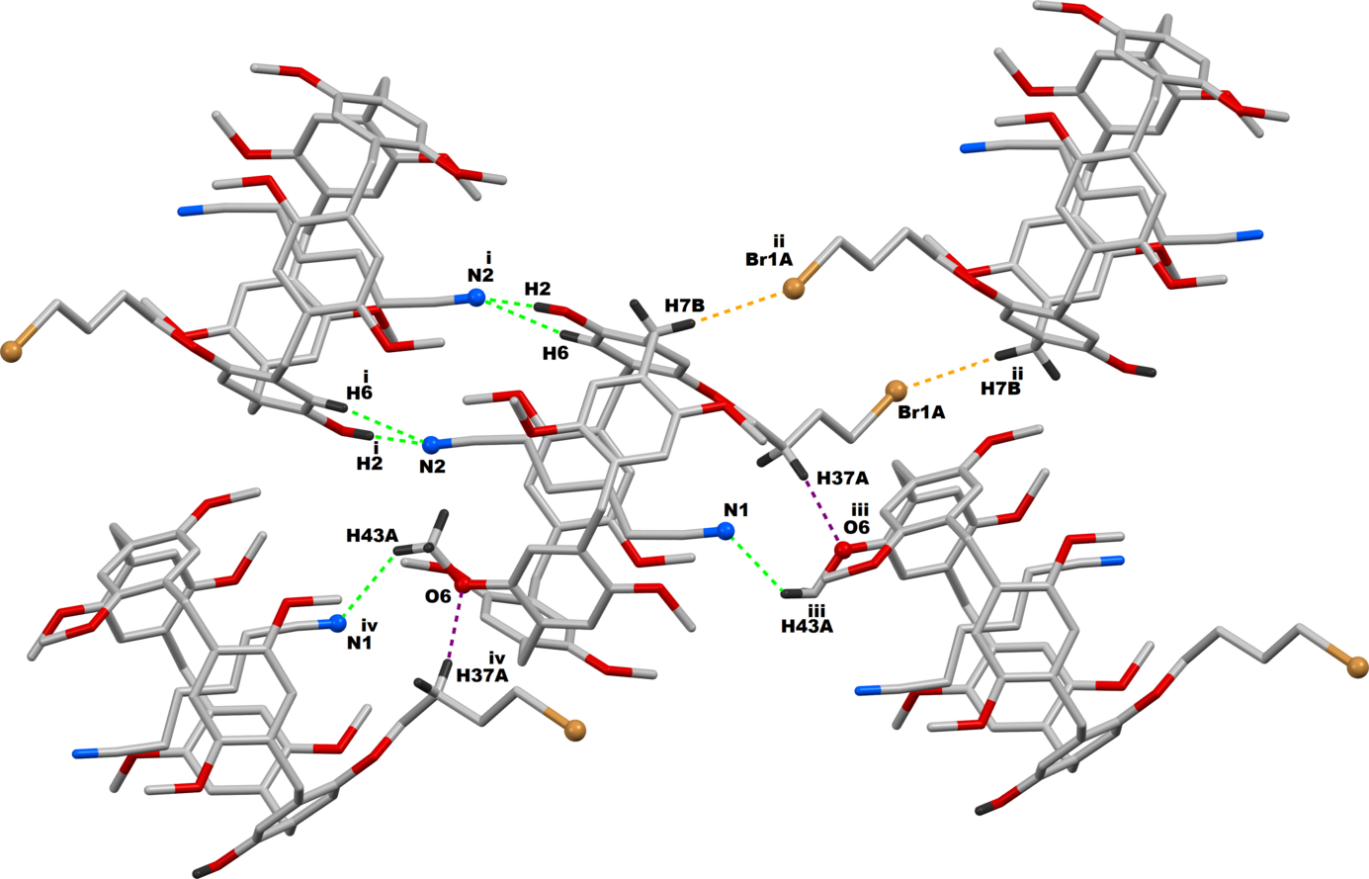
Inter­molecular inter­actions experienced by a given pillar[5]arene mol­ecule involving its neighbouring counterparts; Symmetry code: (i) 1 − *x*, 1 − *y*, 2 − *z*; (ii) 1 − *x*, −*y*, 2 − *z*; (iii) 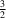 − *x*, −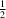 + *y*, 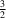 − *z*; (iv) 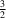 − *x*, 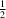 + *y*, 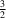 − *z*.

Efficient supra­molecular inter­actions are present in the crystal network of **PilButBrOH·ADN**. Both the hy­droxy and bromo moieties of this difunctionalized pillar[5]arene are engaged in supra­molecular inter­actions with its neighboring counterparts, as demonstrated in Fig. 3 ▸. The OH functional group of **PilButBrOH** (pillar[5]arene **A**) is found to be inter­acting with a nitro­gen atom of the entrapped adipo­nitrile mol­ecule of the adjacent macrocycle (pillar[5]arene **B**). The OH functional group of this pillar[5]arene **B**, in turn, inter­acts with the nitro­gen atom of the adipo­nitrile mol­ecule that is encapsulated within the pillar[5]arene (**A**). As a result, a 1:1 pillar[5]arene (**A**)-pillar[5]arene (**B**) supra­molecular dimer is formed through hydroxyl-mediated inter­action in the crystal structure. The 4-bromo­but­oxy functional group in pillar[5]arene (**A**), on the other hand, inter­acts with the periphery of another **PilButBrOH** mol­ecule (pillar[5]arene **C**) from a different asymmetric unit by a Br⋯H—C inter­action. As in the case of the hy­droxy group inter­actions, the 4-bromo­but­oxy moiety of this pillar[5]arene (**C**) inter­acts with the periphery of pillar[5]arene (**A**) through a second Br⋯H—C inter­action. These two complementary Br⋯H—C inter­actions enable the parent pillar[5]arene (**A**) to behave as a 1:1 pillar[5]arene (**A**)–pillar[5]arene (**C**) supra­molecular dimer. Qu­anti­tative details of the hy­droxy- and bromo­but­oxy-based supra­molecular inter­actions observed in the **PilButBrOH·ADN** systems are given in Table 2 ▸. In addition to the hy­droxy-mediated supra­molecular inter­actions, two complementary N⋯H—C inter­actions are also observed between pillar[5]arene **A** and pillar[5]arene **B**, involving the same nitro­gen atoms of the adipo­nitrile guests as demonstrated in Fig. 4 ▸. On the whole, the terminal nitro­gen atoms of the adipo­nitrile guest are bonded to two neighboring pillar[5]arene mol­ecules from two different asymmetric units through one N⋯O—H and two N⋯H—C inter­actions and thus contribute significantly to the supra­molecular inter­actions of this crystal network. In addition to the N⋯O—H and N⋯H—C mentioned above, a second N⋯H—C inter­action is observed between the adipo­nitrile guest in one pillar[5]arene and a meth­oxy moiety of an adjacent pillar[5]arene mol­ecule from a different asymmetric unit, as illustrated in Fig. 4 ▸. Finally, there is an inter­molecular C—H⋯O bond between a methyl moiety of the pillar[5]arene and an oxygen atom of a meth­oxy-oxygen of a neighbouring pillar[5]arene, which is also depicted in Fig. 4 ▸. The qu­anti­tative details of all these inter­molecular supra­molecular inter­actions are given in Table 2 ▸. As a result of all these supra­molecular inter­actions, each given pillar[5]arene is bonded to four adjacent pillar[5]arene mol­ecules in its crystal network, which are clearly shown in Fig. 4 ▸.

## 4. Hirshfeld surface analysis

**Figure 5:**
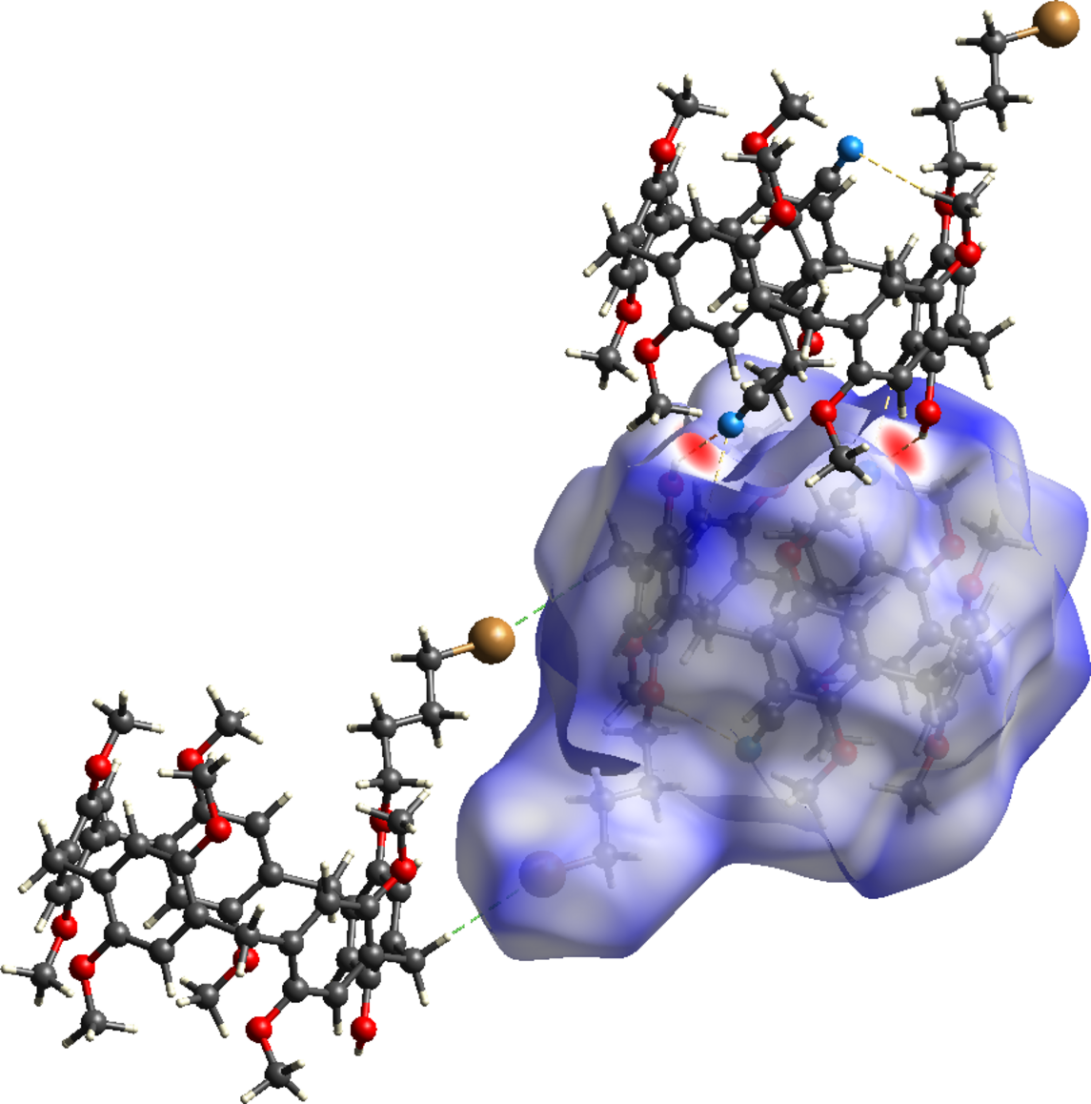
Hirshfeld surfaces (mapped with *d*_norm_) of **PilButBrOH·ADN**. The adjacent pillar[5]arene counterparts are also shown to illustrate the O—H⋯N, C—H⋯N and C—H⋯Br inter­actions.

A Hirshfeld surface analysis was performed using *CrystalExplorer17* (Turner *et al.*, 2017 ▸). The Hirshfeld surface (**HS**) mapped with the *d*_norm_ function for **PilButBrOH·ADN** is shown in Fig. 5 ▸. The strong N⋯H—O supra­molecular inter­actions between the hy­droxy fraction of a given pillar[5]arene mol­ecule and an adipo­nitrile guest belonging to its adjacent pillar[5]arene counterpart appear as intense red spots in the **HS** diagram. Inter­molecular C—H⋯Br inter­actions are shown in this figure as white regions. From the 2D fingerprint plots (McKinnon *et al.*, 2007 ▸), the major inter­molecular inter­actions in the **PilButBrOH·ADN** system are H⋯H (60.6%), C⋯H/H⋯C (16.1%), Br⋯H/H⋯Br (8.0%), O⋯H/H⋯O (7.0%) and, N⋯H/H⋯N (6.8%).

## 5. Database survey

A search in the Cambridge Structural Database (version 5.45, last update June 2024; Groom *et al.*, 2016 ▸) revealed that no A1/A2-difunctionalized pillar[5]arene substituted with a hy­droxy-bromo­but­oxy combination has been reported. The database search showed that the crystal structure of a mono *ortho*-fluoro­phenyl substituted A1/A2-di­hydroxy­pillar[5]arenes has been reported (CASFEL; Wang *et al.*, 2022*b* ▸). While one of hydroxyl groups of the parent pillar[5]arene in this crystal is found to be inter­acting with fluoro­phenyl moiety of an adjacent pillar[5]arene, the other is bonded to the solvent aceto­nitrile. The fluorine atom in this pillar[5]arene also inter­acts with the aceto­nitrile solvent. Similarly, a di­bromo-substituted A1/A2-dihy­droxy pillar[5]arene has also been reported (LIMHEW; Strutt *et al.*, 2013 ▸). As in the case of **PilButBrOH·ADN**, both the hy­droxy as well as the bromine moieties in this pillar[5]arene are engaged in efficient supra­molecular inter­actions with its neighboring pillar[5]arene counterparts. Furthermore, mono *ortho*-allyl-substituted mono­hydroxy­pillar[5]arene has been reported in the literature (VECBAJ; Bojtár *et al.*, 2017 ▸). While the hy­droxy fraction in this pillar[5]arene inter­acts with an adjacent pillar[5]arene *via* a C—H⋯O hydrogen bond, the allyl fraction inter­acts with another pillar[5]arene through a C—H⋯π bond. A non-symmetric pillar[5]arene bearing bromo­but­oxy and proparg­yloxy substitution has been reported as well (VEFQEF; Ding *et al.*, 2017 ▸). In this crystal, the bromo­but­oxy group of the parent pillar[5]arene is bonded to another bromo­but­oxy moiety of a second pillar[5]arene by complementary Br⋯Br inter­actions and the proparg­yloxy group inter­acts with the proparg­yloxy group of a third pillar[5]arene by a C—H⋯π bond.

Many reports on pillar[5]arenes encapsulated with adipo­nitrile or other α,ω-di­cyano­alkanes guests were found in the database in which the supra­molecular inter­actions of the guest species are dependent on the structural details of the host pillar[5]arenes as well as the alkyl chain length of the di­cyano­alkanes. A series of pillar[5]arene-adipo­nitrile host–guest inclusion complexes has been reported in which the pillar[5]arenes are *n*-alk­yloxy (*n*-but­oxy, *n*-pent­yloxy, *n*-hex­yloxy and *n*-hept­yloxy) derivatives (CUDYUY, CUDZIN, CUDZAF and CUDZEJ; Ji *et al.*, 2020 ▸). The adipo­nitrile guest in all these inclusion complexes is located well inside the macrocyclic cavity and does not participate in any supra­molecular inter­actions except for the corresponding host pillar[5]arenes. The crystal structures of host–guest inclusion complexes comprising pillar[5]arene-adipo­nitrile systems in which one of the pillar[5]arene *meso*-positions is embedded with aggregation-induced emission luminogens have appeared in the literature (IFIQEX and IFIQIB; Zhang *et al.*, 2023 ▸). In the IFIQIB crystal where (4-bromo­phen­yl)methyl­idene is the emission luminogen embedded to the pillar[5]arene, each end of the adipo­nitrile guest is bonded to an eth­oxy fraction of a different pillar[5]arene mol­ecule and thus forms a supra­molecular polymer network in the crystal. At the same time, in the IFIQEX crystal where 2,7-di­bromo-9*H*-fluoren-9-yl­idene is the embedded luminogen, the adipo­nitrile guest does not participate in any supra­molecular inter­actions, except for the corresponding host pillar[5]arene. Furthermore, the crystal structure of a bis­(pyrazin-2-yl­oxy)hexane functionalized pillar[5]arene host entrapped with adipo­nitrile guest is reported. In this crystal, the adipo­nitrile is also engaged in supra­molecular polymer formation by inter­acting with both ends of its pillar[5]arene neighbors (RESHEF; Yang *et al.*, 2018 ▸). Host–guest inclusion complexes of pillar[5]-bis-thia­crown with various α,ω-di­cyano­alkanes guests including adipo­nitrile have also been reported (CILROH, CILRUN, CILSAU, CILSEY and CILSIC; Lee *et al.*, 2019*b* ▸). It is observed that when the length of the alkyl chain of the di­cyano­alkane increases, they show a higher tendency to be involved in non-bonding inter­actions with other pillar[5]arenes. The crystal structure of a novel tricylic host mol­ecule consisting of two pillar[5]arene units and a crown ether ring, which selectively binds an adipo­nitrile guest mol­ecule in one pillar[5]arene cavity, has also been reported (SULJIU; Hu *et al.*, 2015 ▸). This entrapped adipo­nitrile is engaged in supra­molecular inter­actions with an adjacent pillar[5]arene through one of its nitrile ends. The crystal structure of an A1/A2-thio­pyridyl pillar[5]arene with an encapsulated 1,8-di­cyano­octane guest is reported where one end of the guest species is inter­acted with an adjacent pillar[5]arene. Furthermore, the combination of this host–guest system with silver(I) ion afforded a diperiodic poly-pseudo-rotaxane (DOQZAN and DOQZOB; Lee *et al.*, 2019*a* ▸). The 1,8-di­cyano­octane guest in this poly-pseudo-rotaxane also participates in supra­molecular inter­actions with the thio­pyridyl moiety of a neighboring pillar[5]arene as well as with the tri­fluoro acetate anion present in the crystal. The asymmetric unit of this crystal contains another 1,8-di­cyano­octane mol­ecule that is not encapsulated by any pillar[5]arene macrocycle. Both terminals of this di­nitrile mol­ecule are involved in CN⋯Ag bonds with the Ag^I^ ion of the complex to complete the formation of the crystal network. A novel A1/A2-thio­pyridyl pillar[5]arene host and 1,8-di­cyano­octane guest yielded a monoperiodic poly-pseudo-rotaxane with HgCl_2_ and its crystal structure has also been published (TECZAG; Kim *et al.*, 2022 ▸). The 1,8-di­cyano­octane guest does not really contribute to the supra­molecular inter­actions in this crystal network except for a single CN⋯H—C inter­action with an adjacent pillar[5]arene.

## 6. Synthesis and crystallization

The synthesis and characterization of **PilButBrOH** has been described earlier (Al-Azemi & Vinodh, 2021 ▸) and is as follows. The first step is the synthesis of A1/A2-bromo­but­oxy-benz­yloxy difunctionalized pillar[5]arene by the co-condensation method (Al-Azemi & Vinodh, 2021 ▸). The benz­yloxy functional group was converted to the hy­droxy derivatives by catalytic hydrogenation (**PilButBrOH**). NMR data of **PilButBrOH**: ^1^H NMR (600 MHz, CDCl_3_) δ: 1.60 (*m*, 4H), 3.20 (*m*, 2H), 3.59 (*m*, 2H), 3.64 (*s*, 4H), 3.73 (*m*, 14H), 3.75 (*m*, 6H), 3.79 (*m*, 10H) 6.68 (*m*, 4H), 6.81 (*s*, 2H), 6.83 (*s*, 2H), 6.85 ppm (*s*, 2H). ^13^C NMR (150 MHz, CDCl_3_) δ: 28.1, 28.5, 28.8, 29.2, 29.6, 29.6, 29.9, 30.3, 31.1, 33.5, 55.6, 55.9, 56.0, 56.1, 56.2, 56.5, 56.6, 68.0, 113.3, 113.8, 113.9, 114.2, 114.4, 114.5, 114.8, 119.1, 123.6, 125.3, 127.1, 128.0, 128.0, 128.3, 128.4, 128.5, 128.8, 129.5, 129.6, 130.0, 133.6, 146.7, 147.7, 148.8, 150.3, 150.9, 150.9, 151.0, 151.0, 151.1, 151.2, 151.2, 152.0 ppm.

Colorless blocks of **PilButBrOH·ADN** crystals suitable for single crystal analysis were grown by dissolving **PilButBrOH** (25 mg) in chloro­form:adipo­nitrile solvent mixture (90:10 *v*/*v*, 1 mL) and subjected to slow solvent evaporation. NMR data of **PilButBrOH·ADN (**1:1 molar equivalent): ^1^H NMR (600 MHz, CDCl_3_) δ: 1.67 (*m*, 4H), 1.90 (*m*, 4H), 2.16 (*m*, 4H), 3.40 (*m*, 2H), 3.59 (*s*, 2H), 3.66 (*m*, 2H), 3.70 (*s*, 4H), 3.81 (*m*, 28H), 6.71 (*s*, 2H), 6.75 (*s*, 2H), 6.89 (*s*, 2H), 6.93 ppm (*s*, 4H). ^13^C NMR (150 MHz, CDCl_3_) δ: 16.7, 24.4, 27.9, 29.4, 29.4, 29.5, 31.4, 33.8, 55.7, 55.8, 55.8, 56.0, 62.2, 113.4, 113.6, 113.9, 118.9, 123.6, 128.2, 128.7, 129.7, 113.0, 146.9, 150.6, 150.6, 151.1, 188.6 ppm.

## 7. Refinement

Crystal data, data collection and structure refinement details are summarized in Table 3 ▸. During the refinement, we noticed that the catalytic reductive de­benzyl­ation used to prepare the **PilButBrOH** caused an undesired side reaction, and in ∼18.5% of the bromo­but­oxy spacers, the bromine was replaced by a hydrogen atom, leading to a simple but­oxy side chain. This required a refining disorder in disorder because the side chain is disordered over three positions (79.74:6.53:13.73%), where the main position further splits between 18.46% of *n*-BuO and 61.28% of 4-BrBuO fractions. This led to a 2.4% lower *R*_1_ value and a significant drop in the residuals. The three parts of the bromo­but­oxy chain were placed into PART 1– PART 3, and each part was assigned a different free variable (FV). Therefore, PART 1 was assigned FV2, PART2 FV3, and PART 3 used FV4. Thereafter, the H2Br substituents of C39*A* were placed in PART 1 and assigned FV 6, while three hydrogen atoms assigned to the same C39*A* atom corresponding to the de-brominated *n*-BuO chain were placed into PART 4 and assigned FV7. SUMP 1 0.00001 1 2 1 3 1 4 was used to constrain the occupancy of the three side chains to 1, while SUMP 0 0.00001 − 1 2 1 6 1 7 was used to ensure that the sum of the FV6 and FV7 was equal to FV2. BIND 1 4 was used to resolve the connectivity around C39*A*. FV 5 was used to refine the proportion of the two positions (62.5:37.5%) of the disordered enclosed adipo­nitrile mol­ecule and FV8 for a similar refinement of a disordered O–CH_3_ methyl group (92.36:7.64% proportion). Additionally, *SIMU, RIGU* and *EADP* were used to restrain/constrain the thermal displacement parameters of the disordered atoms, and *SAME*, *SADI* and *DFIX* were used to adjust the geometry of the disordered fragments. After this refinement, there was still residual electron density around the 4-BrBuO side chain, but any further refinement was unsuccessful, and as the highest peak is 0.4 e Å^−3^, it was deemed unnecessary. For the sake of clarity, only the positions with the largest occupancy for all three disordered groups were used in the above discussions: 4-BrBuO (O1*A*, C36*A*, C37*A*, C38*A*, C39*A*, H39*A*, H39*B*, Br1*A*); adipo­nitrile (N1, C48, C49, C50, C51, C52, C53, N2) and OMe (C41).

All carbon-bound hydrogen atoms were positioned geometrically with C—H distances for methyl, methyl­ene, aromatic H atoms being 0.96, 0.97 and 0.93 Å, respectively, and the thermal factors of hydrogen atoms were refined with *U*_iso_(H) = 1.2*U*_eq_(C), except for hydrogen atoms from methyl groups, where *U*_iso_(H) = 1.5*U*_eq_(C) was used. The acidic proton H2 from the OH group was located from the residual electron-density map and refined with *U*_iso_(H2) = 1.5*U*_eq_(O2). No distance restraints were necessary in this case, as the O—H bond length refined to 0.89 (4) Å.

## Supplementary Material

Crystal structure: contains datablock(s) I. DOI: 10.1107/S2056989024009216/jq2036sup1.cif

Structure factors: contains datablock(s) I. DOI: 10.1107/S2056989024009216/jq2036Isup3.hkl

Supporting information file. DOI: 10.1107/S2056989024009216/jq2036Isup4.mol

NMR Data. DOI: 10.1107/S2056989024009216/jq2036sup5.doc

CCDC reference: 2357919

Additional supporting information:  crystallographic information; 3D view; checkCIF report

## Figures and Tables

**Table 1:** Non-bonding inter­actions (Å, °) between the pillar[5]arene host and adipo­nitrile guest π1–π4 are the centroids of the phenyl rings C1–C6, C8–C13, C15–C20 and C22–C27 respectively.

*D*—H⋯*A*	*D*—H	H⋯*A*	*D*⋯*A*	*D*—H⋯*A*
C49—H49*A*⋯π3	0.99	2.88	3.840 (9)	163
C49—H49*B*⋯O9	0.99	3.29	4.190 (8)	152
C50—H50*A*⋯π1	0.99	3.02	3.812 (9)	137
C50—H50*B*⋯π2	0.99	2.74	3.726 (9)	177
C51—H51*A*⋯π4	0.99	3.18	3.822 (9)	124
C51—H51*B*⋯O6	0.99	3.25	4.207 (9)	164
C52—H52*A*⋯O2	0.99	3.21	3.593 (9)	105
C52—H52*B*⋯π1	0.99	3.11	3.957 (9)	145

**Table 2:** Non-bonding inter­actions(Å, °) among adjacent pillar[5]arenes in **PilButBrOH·ADN** systems

*D*—H⋯*A*	*D*—H	H⋯*A*	*D*⋯*A*	*D*—H⋯*A*
O2—H2⋯N2^i^	0.89 (4)	1.98 (4)	2.866 (10)	178 (4)
C6—H6⋯N2^i^	0.95	2.73	3.46 (1)	134
C7—H7*B*⋯Br1*A*^ii^	0.99	2.950	3.906 (3)	162
C37*A*—H37*A*⋯O6^iii^	0.99	2.60	3.587 (5)	172
C43—H43*A*⋯N1^iv^	0.98	2.77	3.59 (1)	141

Symmetry codes: (i) 1 − *x*, 1 − *y*, 2 − *z*; (ii) 1 − *x*, −*y*, 2 − *z*; (iii) 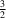 − *x*, −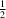 + *y*, 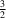 − *z*; (iv) 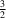 − *x*, 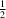 + *y*, 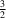 − *z*.

**Table 3:** Experimental details

Crystal data	
Chemical formula	C_47_H_53.18_Br_0.82_O_10_·C_6_H_8_N_2_
*M*_r_	951.38
Crystal system, space group	Monoclinic, *P*2_1_/*n*
Temperature (K)	150
*a*, *b*, *c* (Å)	11.9686 (12), 21.180 (2), 20.107 (2)
β (°)	92.659 (7)
*V* (Å^3^)	5091.5 (9)
*Z*	4
Radiation type	Mo *K*α
μ (mm^−1^)	0.72
Crystal size (mm)	0.20 × 0.20 × 0.18

Data collection	
Diffractometer	Rigaku R-AXIS RAPID
Absorption correction	Multi-scan (*ABSCOR*; Higashi, 1995 ▸)
*T*_min_, *T*_max_	0.403, 0.853
No. of measured, independent and observed [*I* > 2σ(*I*)] reflections	46597, 8895, 5350
*R*_int_	0.053
(sin θ/λ)_max_ (Å^−1^)	0.594

Refinement	
*R*[*F*^2^ > 2σ(*F*^2^)], *wR*(*F*^2^), *S*	0.054, 0.170, 1.02
No. of reflections	8895
No. of parameters	797
No. of restraints	1090
H-atom treatment	H atoms treated by a mixture of independent and constrained refinement
Δρ_max_, Δρ_min_ (e Å^−3^)	0.39, −0.23

Computer programs: *CrystalClear* (Rigaku, 2016 ▸), *CrystalStructure* (Rigaku, 2017 ▸), *SHELXL2019/2* (Sheldrick, 2015 ▸) and *Mercury* (Macrae *et al.*, 2020 ▸).

## supplementary crystallographic information

### A1/A2-Bromobutoxy–hydroxy difunctionalized pillar[5]arene– hexanedinitrile (1/1) Crystal data

**Table:**

C_47_H_53.18_Br_0.82_O_10_·C_6_H_8_N_2_	*F*(000) = 2007
*M**_r_* = 951.38	*D*_x_ = 1.241 Mg m^−^^3^
Monoclinic, *P*2_1_/*n*	Mo *K*α radiation, λ = 0.71075 Å
*a* = 11.9686 (12) Å	Cell parameters from 22410 reflections
*b* = 21.180 (2) Å	θ = 3.0–25.0°
*c* = 20.107 (2) Å	µ = 0.72 mm^−^^1^
β = 92.659 (7)°	*T* = 150 K
*V* = 5091.5 (9) Å^3^	Block, colorless
*Z* = 4	0.20 × 0.20 × 0.18 mm

### A1/A2-Bromobutoxy–hydroxy difunctionalized pillar[5]arene– hexanedinitrile (1/1) Data collection

**Table:**

Rigaku R-AXIS RAPID diffractometer	5350 reflections with *I* > 2σ(*I*)
Detector resolution: 10.000 pixels mm^-1^	*R*_int_ = 0.053
ω scans	θ_max_ = 25.0°, θ_min_ = 3.1°
Absorption correction: multi-scan (ABSCOR; Higashi, 1995)	*h* = −13→14
*T*_min_ = 0.403, *T*_max_ = 0.853	*k* = −25→25
46597 measured reflections	*l* = −23→23
8895 independent reflections	

### A1/A2-Bromobutoxy–hydroxy difunctionalized pillar[5]arene– hexanedinitrile (1/1) Refinement

**Table:**

Refinement on *F*^2^	1090 restraints
Least-squares matrix: full	Hydrogen site location: mixed
*R*[*F*^2^ > 2σ(*F*^2^)] = 0.054	H atoms treated by a mixture of independent and constrained refinement
*wR*(*F*^2^) = 0.170	*w* = 1/[σ^2^(*F*_o_^2^) + (0.0926*P*)^2^ + 0.6834*P*] where *P* = (*F*_o_^2^ + 2*F*_c_^2^)/3
*S* = 1.02	(Δ/σ)_max_ < 0.001
8895 reflections	Δρ_max_ = 0.39 e Å^−^^3^
797 parameters	Δρ_min_ = −0.23 e Å^−^^3^

### A1/A2-Bromobutoxy–hydroxy difunctionalized pillar[5]arene– hexanedinitrile (1/1) Special details

**Table:**

Geometry. All esds (except the esd in the dihedral angle between two l.s. planes) are estimated using the full covariance matrix. The cell esds are taken into account individually in the estimation of esds in distances, angles and torsion angles; correlations between esds in cell parameters are only used when they are defined by crystal symmetry. An approximate (isotropic) treatment of cell esds is used for estimating esds involving l.s. planes.
Refinement. The single-crystal data were collected on Rigaku Rapid II diffractometer using Mo*K_α_* radiation at 150 K. The data were processed by ′CrystalClear′ software package (Rigaku, 2016). The structure was solved by direct methods using the ′CrystalStructure′ crystallographic software package (Rigaku, 2017) and the refinement was performed using SHELXL2019/2 (Sheldrick 2015).

### A1/A2-Bromobutoxy–hydroxy difunctionalized pillar[5]arene– hexanedinitrile (1/1) Fractional atomic coordinates and isotropic or equivalent isotropic displacement parameters (Å^2^)

**Table:**

	*x*	*y*	*z*	*U*_iso_*/*U*_eq_	Occ. (<1)
O1A	0.6290 (3)	0.16658 (14)	0.9994 (3)	0.0801 (12)	0.7974 (18)
C36A	0.6399 (4)	0.12395 (19)	0.9457 (2)	0.0924 (11)	0.7974 (18)
H36A	0.650499	0.147636	0.903999	0.111*	0.7974 (18)
H36B	0.571359	0.097991	0.939611	0.111*	0.7974 (18)
C37A	0.7409 (3)	0.08159 (17)	0.9615 (2)	0.0946 (11)	0.7974 (18)
H37A	0.764911	0.062328	0.919651	0.113*	0.7974 (18)
H37B	0.803342	0.107934	0.979815	0.113*	0.7974 (18)
C38A	0.7186 (4)	0.03050 (17)	1.0098 (2)	0.0943 (11)	0.7974 (18)
H38A	0.655068	0.004602	0.992045	0.113*	0.7974 (18)
H38B	0.696536	0.049642	1.052126	0.113*	0.7974 (18)
C39A	0.8194 (4)	−0.0120 (2)	1.0235 (3)	0.1039 (12)	0.7974 (18)
H39C	0.800416	−0.044823	1.055529	0.156*	0.185 (2)
H39D	0.840585	−0.031888	0.981949	0.156*	0.185 (2)
H39E	0.882060	0.013160	1.042042	0.156*	0.185 (2)
H39A	0.882816	0.013282	1.042305	0.125*	0.613 (2)
H39B	0.842215	−0.031297	0.981418	0.125*	0.613 (2)
Br1A	0.78238 (16)	−0.07848 (6)	1.08639 (10)	0.1313 (6)	0.613 (2)
C2	0.5502 (2)	0.21390 (12)	0.99377 (14)	0.0638 (6)	
O1B	0.624 (2)	0.1640 (10)	0.9986 (19)	0.088 (2)	0.0653 (18)
C36B	0.579 (3)	0.1058 (15)	1.019 (3)	0.0906 (19)	0.0653 (18)
H36C	0.524773	0.089375	0.984859	0.109*	0.0653 (18)
H36D	0.538763	0.111889	1.061026	0.109*	0.0653 (18)
C37B	0.674 (3)	0.0589 (15)	1.031 (2)	0.0951 (18)	0.0653 (18)
H37C	0.693953	0.040168	0.988283	0.114*	0.0653 (18)
H37D	0.740998	0.081463	1.050136	0.114*	0.0653 (18)
C38B	0.643 (3)	0.0064 (16)	1.079 (2)	0.101 (2)	0.0653 (18)
H38C	0.569503	−0.011058	1.064222	0.121*	0.0653 (18)
H38D	0.637271	0.024094	1.123931	0.121*	0.0653 (18)
C39B	0.730 (3)	−0.0466 (16)	1.081 (3)	0.103 (2)	0.0653 (18)
H39F	0.695006	−0.082283	1.055912	0.124*	0.0653 (18)
H39G	0.737403	−0.060307	1.128092	0.124*	0.0653 (18)
Br1B	0.8751 (12)	−0.0392 (6)	1.0518 (8)	0.157 (4)	0.0653 (18)
O1C	0.610 (2)	0.1593 (6)	1.0088 (14)	0.089 (2)	0.137 (2)
C36C	0.5682 (19)	0.1004 (7)	0.9850 (14)	0.0909 (18)	0.137 (2)
H36G	0.557441	0.101303	0.935927	0.109*	0.137 (2)
H36H	0.495352	0.090996	1.004264	0.109*	0.137 (2)
C37C	0.6552 (18)	0.0497 (8)	1.0064 (14)	0.0963 (18)	0.137 (2)
H37E	0.705832	0.044146	0.969300	0.116*	0.137 (2)
H37F	0.700855	0.066859	1.044556	0.116*	0.137 (2)
C38C	0.6152 (16)	−0.0155 (8)	1.0262 (14)	0.102 (2)	0.137 (2)
H38E	0.557749	−0.028763	0.992022	0.123*	0.137 (2)
H38F	0.576759	−0.010603	1.068400	0.123*	0.137 (2)
C39C	0.6982 (17)	−0.0711 (9)	1.0359 (14)	0.104 (2)	0.137 (2)
H39H	0.719706	−0.084975	0.991229	0.125*	0.137 (2)
H39I	0.657568	−0.106576	1.055778	0.125*	0.137 (2)
Br1C	0.8224 (7)	−0.0586 (4)	1.0850 (4)	0.123 (2)	0.137 (2)
O2	0.32430 (17)	0.36174 (11)	0.97909 (12)	0.0890 (7)	
H2	0.345 (3)	0.392 (2)	1.007 (2)	0.133*	
O3	0.36303 (18)	0.18197 (9)	0.79308 (11)	0.0850 (6)	
O4	0.31980 (16)	0.43675 (9)	0.73885 (12)	0.0880 (6)	
O5	0.58873 (18)	0.29024 (11)	0.59519 (12)	0.0942 (7)	
O6	0.68225 (18)	0.52704 (10)	0.69892 (12)	0.0929 (7)	
O7	0.96315 (19)	0.34123 (13)	0.70008 (11)	0.1027 (7)	
O8	0.92576 (17)	0.51595 (9)	0.90155 (10)	0.0831 (6)	
O9	0.99031 (15)	0.27027 (9)	0.92914 (10)	0.0770 (5)	
O10	0.71311 (16)	0.40323 (10)	1.09101 (11)	0.0843 (6)	
C1	0.5692 (2)	0.26687 (12)	1.03394 (12)	0.0578 (6)	
C3	0.4574 (2)	0.21092 (12)	0.94922 (13)	0.0642 (7)	
H3	0.446014	0.174378	0.922357	0.077*	
C4	0.38090 (19)	0.26075 (12)	0.94341 (12)	0.0581 (6)	
C5	0.3994 (2)	0.31285 (13)	0.98418 (14)	0.0633 (7)	
C6	0.4921 (2)	0.31522 (13)	1.02912 (13)	0.0632 (7)	
H6	0.502177	0.351119	1.057085	0.076*	
C7	0.2827 (2)	0.25796 (14)	0.89323 (13)	0.0657 (7)	
H7A	0.219818	0.282628	0.910294	0.079*	
H7B	0.257697	0.213594	0.888113	0.079*	
C8	0.31140 (18)	0.28380 (12)	0.82541 (13)	0.0581 (6)	
C9	0.3536 (2)	0.24494 (12)	0.77650 (14)	0.0623 (7)	
C10	0.3833 (2)	0.26966 (13)	0.71589 (14)	0.0650 (7)	
H10	0.412121	0.242392	0.683312	0.078*	
C11	0.37159 (19)	0.33375 (13)	0.70209 (13)	0.0597 (7)	
C12	0.32887 (19)	0.37222 (12)	0.75059 (14)	0.0620 (7)	
C13	0.29948 (19)	0.34732 (12)	0.81074 (14)	0.0627 (7)	
H13	0.270159	0.374657	0.843089	0.075*	
C14	0.4071 (2)	0.36041 (14)	0.63637 (13)	0.0712 (7)	
H14A	0.355321	0.394869	0.622366	0.085*	
H14B	0.400995	0.326889	0.602080	0.085*	
C15	0.5260 (2)	0.38577 (13)	0.63998 (13)	0.0644 (7)	
C16	0.6152 (2)	0.34916 (14)	0.62035 (13)	0.0705 (7)	
C17	0.7242 (2)	0.37320 (16)	0.62685 (13)	0.0755 (8)	
H17	0.784561	0.348427	0.612307	0.091*	
C18	0.7458 (2)	0.43200 (15)	0.65388 (13)	0.0708 (8)	
C19	0.6564 (2)	0.46855 (14)	0.67296 (14)	0.0701 (7)	
C20	0.5483 (2)	0.44537 (13)	0.66553 (14)	0.0679 (7)	
H20	0.487826	0.471059	0.678307	0.082*	
C21	0.8663 (2)	0.45534 (18)	0.66408 (14)	0.0849 (9)	
H21A	0.914090	0.433802	0.632229	0.102*	
H21B	0.869131	0.501278	0.655220	0.102*	
C22	0.9104 (2)	0.44229 (16)	0.73460 (14)	0.0718 (8)	
C23	0.9576 (2)	0.38387 (16)	0.75118 (14)	0.0745 (8)	
C24	0.9968 (2)	0.37175 (15)	0.81623 (14)	0.0700 (7)	
H24	1.031625	0.332414	0.826518	0.084*	
C25	0.9860 (2)	0.41602 (13)	0.86613 (13)	0.0615 (7)	
C26	0.9373 (2)	0.47422 (13)	0.85017 (14)	0.0651 (7)	
C27	0.9021 (2)	0.48698 (15)	0.78402 (14)	0.0719 (8)	
H27	0.871791	0.527304	0.773017	0.086*	
C28	1.0249 (2)	0.40075 (13)	0.93719 (13)	0.0616 (7)	
H28A	1.045515	0.440415	0.960741	0.074*	
H28B	1.092461	0.373846	0.936705	0.074*	
C29	0.93527 (19)	0.36686 (12)	0.97484 (12)	0.0558 (6)	
C30	0.91911 (19)	0.30210 (12)	0.96954 (12)	0.0573 (6)	
C31	0.8354 (2)	0.27243 (12)	1.00360 (12)	0.0580 (6)	
H31	0.825188	0.228128	0.999134	0.070*	
C32	0.76635 (19)	0.30670 (12)	1.04419 (12)	0.0563 (6)	
C33	0.7833 (2)	0.37116 (13)	1.05013 (13)	0.0610 (6)	
C34	0.8661 (2)	0.40060 (13)	1.01550 (13)	0.0611 (6)	
H34	0.875820	0.444963	1.019692	0.073*	
C35	0.6736 (2)	0.27279 (13)	1.07921 (13)	0.0639 (7)	
H35A	0.699691	0.230184	1.092965	0.077*	
H35B	0.655725	0.296497	1.119790	0.077*	
C40	0.4037 (4)	0.13995 (15)	0.7448 (2)	0.1170 (13)	
H40A	0.410223	0.097424	0.763809	0.175*	
H40B	0.477344	0.154233	0.731587	0.175*	
H40C	0.351694	0.139182	0.705724	0.175*	
C41	0.2117 (3)	0.46022 (18)	0.7290 (2)	0.1021 (14)	0.924 (6)
H41A	0.170966	0.434965	0.694951	0.153*	0.924 (6)
H41B	0.214907	0.504254	0.714200	0.153*	0.924 (6)
H41C	0.172973	0.457976	0.770806	0.153*	0.924 (6)
C41A	0.278 (4)	0.4748 (12)	0.7877 (15)	0.1021 (14)	0.076 (6)
H41D	0.220495	0.451679	0.810934	0.153*	0.076 (6)
H41E	0.244799	0.512832	0.767147	0.153*	0.076 (6)
H41F	0.338605	0.486876	0.819496	0.153*	0.076 (6)
C42	0.6756 (3)	0.25247 (19)	0.5720 (2)	0.1175 (13)	
H42A	0.728380	0.242243	0.609163	0.176*	
H42B	0.714638	0.275492	0.537782	0.176*	
H42C	0.644114	0.213369	0.552992	0.176*	
C43	0.5938 (4)	0.56629 (17)	0.7188 (3)	0.1271 (15)	
H43A	0.624596	0.606040	0.736633	0.191*	
H43B	0.552693	0.544818	0.753306	0.191*	
H43C	0.542938	0.575135	0.680366	0.191*	
C44	1.0073 (3)	0.2809 (2)	0.7151 (2)	0.1240 (15)	
H44A	0.999801	0.254001	0.675511	0.186*	
H44B	0.966173	0.261987	0.751158	0.186*	
H44C	1.086475	0.284786	0.729159	0.186*	
C45	0.8753 (3)	0.57512 (15)	0.88739 (18)	0.0958 (10)	
H45A	0.801195	0.568578	0.865718	0.144*	
H45B	0.922067	0.599197	0.857699	0.144*	
H45C	0.867760	0.598595	0.928942	0.144*	
C46	0.9785 (3)	0.20465 (15)	0.9228 (2)	0.0969 (10)	
H46A	0.902734	0.194691	0.905415	0.145*	
H46B	0.991513	0.184781	0.966509	0.145*	
H46C	1.033093	0.188620	0.892113	0.145*	
C47	0.7317 (3)	0.46807 (16)	1.1017 (2)	0.1099 (12)	
H47A	0.720997	0.490778	1.059422	0.165*	
H47B	0.808300	0.474616	1.119713	0.165*	
H47C	0.678705	0.483998	1.133428	0.165*	
N1	0.6884 (13)	0.1826 (4)	0.7681 (6)	0.153 (4)	0.625 (8)
C48	0.7035 (17)	0.2360 (4)	0.7776 (7)	0.126 (2)	0.625 (8)
C49	0.7166 (6)	0.3028 (3)	0.7960 (5)	0.120 (2)	0.625 (8)
H49A	0.713396	0.328781	0.755032	0.144*	0.625 (8)
H49B	0.791025	0.309049	0.818579	0.144*	0.625 (8)
C50	0.6293 (7)	0.3247 (3)	0.8404 (5)	0.105 (2)	0.625 (8)
H50A	0.633032	0.300413	0.882539	0.127*	0.625 (8)
H50B	0.554041	0.318957	0.818674	0.127*	0.625 (8)
C51	0.6521 (7)	0.3963 (3)	0.8546 (5)	0.106 (2)	0.625 (8)
H51A	0.727090	0.401772	0.876720	0.127*	0.625 (8)
H51B	0.649674	0.420260	0.812291	0.127*	0.625 (8)
C52	0.5652 (7)	0.4198 (3)	0.8981 (5)	0.124 (2)	0.625 (8)
H52A	0.491540	0.418426	0.873661	0.148*	0.625 (8)
H52B	0.561603	0.391966	0.937502	0.148*	0.625 (8)
C53	0.5886 (14)	0.4848 (4)	0.9202 (6)	0.120 (2)	0.625 (8)
N2	0.610 (2)	0.5372 (5)	0.9327 (8)	0.135 (4)	0.625 (8)
N1B	0.710 (2)	0.1922 (8)	0.7416 (8)	0.141 (5)	0.375 (8)
C48B	0.702 (3)	0.2278 (8)	0.7850 (11)	0.126 (3)	0.375 (8)
C49B	0.7096 (12)	0.2804 (6)	0.8328 (8)	0.124 (2)	0.375 (8)
H49C	0.690537	0.264687	0.877251	0.149*	0.375 (8)
H49D	0.787609	0.296010	0.836214	0.149*	0.375 (8)
C50B	0.6352 (14)	0.3327 (6)	0.8139 (7)	0.109 (3)	0.375 (8)
H50C	0.556833	0.317813	0.809341	0.131*	0.375 (8)
H50D	0.656074	0.350849	0.770884	0.131*	0.375 (8)
C51B	0.6481 (14)	0.3837 (6)	0.8706 (8)	0.110 (3)	0.375 (8)
H51C	0.636013	0.364028	0.914362	0.132*	0.375 (8)
H51D	0.724370	0.401802	0.871721	0.132*	0.375 (8)
C52B	0.5655 (11)	0.4332 (5)	0.8572 (8)	0.119 (2)	0.375 (8)
H52C	0.489617	0.416165	0.863136	0.142*	0.375 (8)
H52D	0.569423	0.447405	0.810428	0.142*	0.375 (8)
C53B	0.586 (2)	0.4876 (7)	0.9023 (10)	0.122 (3)	0.375 (8)
N2B	0.595 (4)	0.5254 (10)	0.9442 (11)	0.125 (5)	0.375 (8)

### A1/A2-Bromobutoxy–hydroxy difunctionalized pillar[5]arene– hexanedinitrile (1/1) Atomic displacement parameters (Å^2^)

**Table:**

	*U*^11^	*U*^22^	*U*^33^	*U*^12^	*U*^13^	*U*^23^
O1A	0.0864 (19)	0.0704 (15)	0.0815 (19)	0.0161 (14)	−0.0167 (17)	−0.0070 (14)
C36A	0.109 (2)	0.076 (2)	0.091 (2)	0.0240 (19)	−0.014 (2)	−0.0088 (19)
C37A	0.106 (2)	0.082 (2)	0.095 (2)	0.0166 (19)	−0.008 (2)	−0.0168 (19)
C38A	0.109 (2)	0.075 (2)	0.096 (2)	0.0220 (19)	−0.026 (2)	−0.0154 (19)
C39A	0.123 (3)	0.082 (2)	0.104 (3)	0.025 (2)	−0.024 (2)	−0.011 (2)
Br1A	0.1659 (13)	0.0687 (6)	0.1577 (10)	0.0128 (6)	−0.0089 (9)	0.0205 (5)
C2	0.0599 (14)	0.0644 (15)	0.0668 (16)	0.0060 (12)	−0.0001 (13)	0.0072 (13)
O1B	0.097 (4)	0.076 (4)	0.088 (4)	0.018 (4)	−0.013 (4)	−0.007 (4)
C36B	0.102 (3)	0.077 (3)	0.091 (3)	0.017 (3)	−0.015 (3)	−0.009 (3)
C37B	0.109 (3)	0.079 (3)	0.095 (3)	0.020 (3)	−0.019 (3)	−0.009 (3)
C38B	0.116 (4)	0.083 (4)	0.100 (4)	0.019 (4)	−0.021 (4)	−0.009 (4)
C39B	0.120 (4)	0.083 (4)	0.104 (4)	0.024 (4)	−0.024 (4)	−0.008 (4)
Br1B	0.170 (7)	0.137 (6)	0.160 (7)	0.052 (5)	−0.043 (6)	0.011 (6)
O1C	0.099 (4)	0.078 (3)	0.089 (4)	0.020 (3)	−0.014 (4)	−0.004 (3)
C36C	0.102 (3)	0.079 (3)	0.091 (3)	0.017 (3)	−0.014 (3)	−0.006 (3)
C37C	0.110 (3)	0.080 (3)	0.096 (3)	0.020 (3)	−0.019 (3)	−0.009 (3)
C38C	0.117 (4)	0.086 (3)	0.101 (4)	0.019 (3)	−0.020 (4)	−0.011 (3)
C39C	0.123 (4)	0.085 (4)	0.103 (4)	0.017 (4)	−0.023 (4)	−0.009 (4)
Br1C	0.148 (5)	0.113 (5)	0.102 (3)	0.022 (3)	−0.053 (3)	0.013 (3)
O2	0.0690 (12)	0.0927 (15)	0.1039 (17)	0.0229 (11)	−0.0103 (11)	−0.0164 (12)
O3	0.1092 (15)	0.0611 (12)	0.0851 (14)	−0.0004 (10)	0.0072 (12)	0.0008 (10)
O4	0.0700 (12)	0.0691 (13)	0.1262 (19)	0.0058 (10)	0.0201 (12)	0.0198 (12)
O5	0.0907 (15)	0.0946 (15)	0.0968 (16)	−0.0027 (12)	0.0002 (12)	−0.0255 (13)
O6	0.0884 (14)	0.0832 (15)	0.1061 (17)	−0.0238 (12)	−0.0066 (12)	−0.0036 (12)
O7	0.0955 (16)	0.141 (2)	0.0710 (14)	0.0204 (15)	0.0009 (12)	−0.0235 (14)
O8	0.1018 (15)	0.0726 (12)	0.0727 (13)	0.0003 (11)	−0.0176 (11)	−0.0014 (10)
O9	0.0733 (12)	0.0696 (12)	0.0897 (14)	0.0030 (9)	0.0211 (10)	−0.0075 (10)
O10	0.0774 (12)	0.0824 (14)	0.0948 (15)	−0.0006 (10)	0.0237 (11)	−0.0218 (11)
C1	0.0551 (14)	0.0694 (16)	0.0494 (14)	−0.0076 (12)	0.0076 (11)	0.0051 (12)
C3	0.0643 (15)	0.0651 (16)	0.0629 (16)	−0.0047 (13)	0.0007 (13)	0.0001 (13)
C4	0.0454 (13)	0.0715 (16)	0.0581 (15)	−0.0034 (12)	0.0077 (11)	0.0081 (13)
C5	0.0508 (14)	0.0729 (17)	0.0665 (17)	0.0039 (13)	0.0069 (12)	0.0012 (14)
C6	0.0573 (15)	0.0718 (17)	0.0610 (16)	0.0002 (13)	0.0069 (12)	−0.0058 (13)
C7	0.0496 (14)	0.0774 (17)	0.0704 (18)	−0.0031 (12)	0.0053 (12)	0.0044 (14)
C8	0.0400 (12)	0.0714 (17)	0.0624 (16)	−0.0043 (11)	−0.0034 (11)	0.0002 (14)
C9	0.0616 (15)	0.0580 (16)	0.0663 (17)	−0.0066 (12)	−0.0073 (13)	−0.0001 (13)
C10	0.0618 (15)	0.0700 (17)	0.0624 (17)	−0.0061 (13)	−0.0054 (13)	−0.0094 (14)
C11	0.0485 (13)	0.0697 (17)	0.0601 (16)	−0.0111 (12)	−0.0063 (12)	0.0016 (13)
C12	0.0450 (13)	0.0616 (16)	0.0791 (19)	−0.0024 (11)	0.0008 (13)	0.0053 (14)
C13	0.0472 (13)	0.0686 (17)	0.0724 (18)	0.0005 (12)	0.0038 (12)	−0.0025 (14)
C14	0.0646 (16)	0.0861 (19)	0.0617 (17)	−0.0132 (14)	−0.0078 (13)	0.0032 (15)
C15	0.0644 (16)	0.0801 (18)	0.0483 (15)	−0.0102 (14)	−0.0015 (12)	0.0078 (13)
C16	0.0712 (18)	0.086 (2)	0.0543 (16)	−0.0078 (15)	−0.0018 (13)	−0.0027 (14)
C17	0.0666 (17)	0.110 (2)	0.0503 (16)	0.0020 (17)	0.0033 (13)	0.0023 (16)
C18	0.0655 (17)	0.098 (2)	0.0485 (15)	−0.0183 (16)	−0.0021 (13)	0.0108 (15)
C19	0.0722 (18)	0.0805 (19)	0.0568 (16)	−0.0163 (15)	−0.0040 (14)	0.0104 (14)
C20	0.0636 (16)	0.0759 (18)	0.0640 (17)	−0.0056 (14)	0.0000 (13)	0.0058 (14)
C21	0.0647 (17)	0.130 (3)	0.0598 (17)	−0.0223 (17)	−0.0010 (13)	0.0135 (18)
C22	0.0491 (14)	0.108 (2)	0.0584 (17)	−0.0166 (15)	0.0036 (12)	0.0102 (17)
C23	0.0547 (15)	0.111 (2)	0.0580 (18)	−0.0107 (16)	0.0094 (13)	−0.0128 (17)
C24	0.0519 (14)	0.095 (2)	0.0634 (17)	−0.0009 (14)	0.0040 (13)	0.0011 (16)
C25	0.0464 (13)	0.0799 (18)	0.0583 (16)	−0.0140 (13)	0.0032 (11)	0.0018 (14)
C26	0.0556 (14)	0.0728 (18)	0.0663 (18)	−0.0140 (13)	−0.0019 (13)	0.0073 (15)
C27	0.0619 (16)	0.0845 (19)	0.0682 (19)	−0.0200 (14)	−0.0092 (14)	0.0146 (16)
C28	0.0508 (13)	0.0718 (16)	0.0614 (16)	−0.0066 (12)	−0.0047 (12)	0.0032 (13)
C29	0.0482 (13)	0.0664 (16)	0.0521 (14)	−0.0036 (11)	−0.0063 (11)	0.0032 (12)
C30	0.0499 (13)	0.0699 (16)	0.0520 (14)	0.0049 (12)	0.0030 (11)	−0.0019 (12)
C31	0.0593 (14)	0.0590 (14)	0.0551 (15)	0.0004 (12)	−0.0049 (12)	0.0047 (12)
C32	0.0506 (13)	0.0736 (17)	0.0443 (13)	0.0004 (12)	−0.0025 (11)	0.0030 (12)
C33	0.0542 (14)	0.0698 (17)	0.0587 (16)	0.0036 (13)	0.0011 (12)	−0.0059 (13)
C34	0.0557 (14)	0.0658 (15)	0.0615 (16)	−0.0020 (13)	−0.0012 (13)	−0.0059 (13)
C35	0.0570 (14)	0.0820 (18)	0.0525 (15)	−0.0044 (13)	0.0008 (12)	0.0070 (13)
C40	0.166 (4)	0.063 (2)	0.123 (3)	0.001 (2)	0.020 (3)	−0.016 (2)
C41	0.083 (2)	0.091 (3)	0.133 (4)	0.030 (2)	0.026 (2)	0.026 (2)
C41A	0.083 (2)	0.091 (3)	0.133 (4)	0.030 (2)	0.026 (2)	0.026 (2)
C42	0.119 (3)	0.110 (3)	0.122 (3)	0.028 (2)	−0.009 (2)	−0.035 (2)
C43	0.119 (3)	0.080 (2)	0.181 (4)	−0.003 (2)	−0.007 (3)	−0.018 (3)
C44	0.112 (3)	0.166 (4)	0.094 (3)	0.038 (3)	0.002 (2)	−0.055 (3)
C45	0.111 (3)	0.079 (2)	0.095 (2)	0.0090 (18)	−0.018 (2)	0.0075 (18)
C46	0.087 (2)	0.082 (2)	0.124 (3)	0.0067 (17)	0.025 (2)	−0.022 (2)
C47	0.114 (3)	0.084 (2)	0.134 (3)	0.005 (2)	0.028 (2)	−0.031 (2)
N1	0.168 (7)	0.123 (5)	0.169 (8)	0.028 (5)	0.016 (7)	−0.022 (6)
C48	0.122 (4)	0.120 (4)	0.134 (5)	0.014 (4)	−0.001 (4)	−0.015 (4)
C49	0.121 (4)	0.122 (4)	0.119 (4)	−0.009 (3)	0.024 (3)	0.001 (3)
C50	0.099 (3)	0.106 (3)	0.112 (4)	−0.009 (3)	0.013 (3)	−0.008 (3)
C51	0.099 (3)	0.109 (4)	0.111 (4)	−0.009 (3)	0.011 (3)	−0.012 (3)
C52	0.126 (4)	0.110 (3)	0.137 (5)	−0.015 (3)	0.029 (4)	−0.012 (4)
C53	0.120 (4)	0.107 (4)	0.133 (6)	−0.010 (4)	0.011 (5)	−0.026 (4)
N2	0.128 (8)	0.106 (5)	0.171 (7)	−0.005 (5)	0.017 (6)	−0.030 (6)
N1B	0.155 (9)	0.135 (8)	0.135 (9)	0.023 (7)	0.017 (8)	−0.022 (6)
C48B	0.126 (5)	0.120 (5)	0.133 (5)	0.008 (5)	0.001 (5)	−0.015 (5)
C49B	0.122 (4)	0.125 (5)	0.126 (5)	0.004 (4)	−0.003 (5)	−0.006 (4)
C50B	0.103 (4)	0.108 (4)	0.116 (5)	−0.005 (4)	0.008 (5)	−0.009 (4)
C51B	0.102 (4)	0.108 (4)	0.119 (5)	−0.009 (4)	0.015 (4)	−0.012 (4)
C52B	0.120 (4)	0.115 (4)	0.121 (5)	−0.004 (4)	0.003 (5)	−0.015 (4)
C53B	0.120 (5)	0.109 (5)	0.137 (6)	−0.009 (5)	0.008 (6)	−0.029 (4)
N2B	0.128 (10)	0.106 (8)	0.142 (8)	−0.011 (8)	0.004 (8)	−0.028 (6)

### A1/A2-Bromobutoxy–hydroxy difunctionalized pillar[5]arene– hexanedinitrile (1/1) Geometric parameters (Å, º)

**Table:**

O1A—C2	1.377 (3)	C16—C17	1.400 (4)
O1A—C36A	1.419 (5)	C17—C18	1.378 (4)
C36A—C37A	1.527 (4)	C17—H17	0.9500
C36A—H36A	0.9900	C18—C19	1.389 (4)
C36A—H36B	0.9900	C18—C21	1.529 (4)
C37A—C38A	1.486 (4)	C19—C20	1.386 (4)
C37A—H37A	0.9900	C20—H20	0.9500
C37A—H37B	0.9900	C21—C22	1.516 (4)
C38A—C39A	1.521 (4)	C21—H21A	0.9900
C38A—H38A	0.9900	C21—H21B	0.9900
C38A—H38B	0.9900	C22—C27	1.379 (4)
C39A—Br1A	1.956 (5)	C22—C23	1.394 (4)
C39A—H39C	0.9800	C23—C24	1.393 (4)
C39A—H39D	0.9800	C24—C25	1.384 (4)
C39A—H39E	0.9800	C24—H24	0.9500
C39A—H39A	0.9900	C25—C26	1.394 (4)
C39A—H39B	0.9900	C25—C28	1.517 (4)
C2—O1B	1.377 (6)	C26—C27	1.403 (4)
C2—O1C	1.383 (7)	C27—H27	0.9500
C2—C1	1.395 (4)	C28—C29	1.521 (3)
C2—C3	1.395 (3)	C28—H28A	0.9900
O1B—C36B	1.42 (2)	C28—H28B	0.9900
C36B—C37B	1.526 (6)	C29—C34	1.388 (3)
C36B—H36C	0.9900	C29—C30	1.389 (3)
C36B—H36D	0.9900	C30—C31	1.390 (3)
C37B—C38B	1.520 (6)	C31—C32	1.393 (3)
C37B—H37C	0.9900	C31—H31	0.9500
C37B—H37D	0.9900	C32—C33	1.384 (4)
C38B—C39B	1.524 (6)	C32—C35	1.522 (3)
C38B—H38C	0.9900	C33—C34	1.386 (4)
C38B—H38D	0.9900	C34—H34	0.9500
C39B—Br1B	1.868 (18)	C35—H35A	0.9900
C39B—H39F	0.9900	C35—H35B	0.9900
C39B—H39G	0.9900	C40—H40A	0.9800
O1C—C36C	1.418 (17)	C40—H40B	0.9800
C36C—C37C	1.544 (9)	C40—H40C	0.9800
C36C—H36G	0.9900	C41—H41A	0.9800
C36C—H36H	0.9900	C41—H41B	0.9800
C37C—C38C	1.520 (9)	C41—H41C	0.9800
C37C—H37E	0.9900	C41A—H41D	0.9800
C37C—H37F	0.9900	C41A—H41E	0.9800
C38C—C39C	1.546 (9)	C41A—H41F	0.9800
C38C—H38E	0.9900	C42—H42A	0.9800
C38C—H38F	0.9900	C42—H42B	0.9800
C39C—Br1C	1.766 (16)	C42—H42C	0.9800
C39C—H39H	0.9900	C43—H43A	0.9800
C39C—H39I	0.9900	C43—H43B	0.9800
O2—C5	1.372 (3)	C43—H43C	0.9800
O2—H2	0.89 (4)	C44—H44A	0.9800
O3—C9	1.378 (3)	C44—H44B	0.9800
O3—C40	1.419 (4)	C44—H44C	0.9800
O4—C41A	1.382 (10)	C45—H45A	0.9800
O4—C12	1.390 (3)	C45—H45B	0.9800
O4—C41	1.392 (4)	C45—H45C	0.9800
O5—C16	1.378 (3)	C46—H46A	0.9800
O5—C42	1.408 (4)	C46—H46B	0.9800
O6—C19	1.374 (3)	C46—H46C	0.9800
O6—C43	1.419 (4)	C47—H47A	0.9800
O7—C23	1.372 (3)	C47—H47B	0.9800
O7—C44	1.410 (5)	C47—H47C	0.9800
O8—C26	1.372 (3)	N1—C48	1.160 (6)
O8—C45	1.414 (3)	C48—C49	1.469 (6)
O9—C30	1.380 (3)	C49—C50	1.481 (6)
O9—C46	1.402 (3)	C49—H49A	0.9900
O10—C33	1.381 (3)	C49—H49B	0.9900
O10—C47	1.406 (4)	C50—C51	1.564 (6)
C1—C6	1.379 (3)	C50—H50A	0.9900
C1—C35	1.517 (3)	C50—H50B	0.9900
C3—C4	1.399 (3)	C51—C52	1.476 (6)
C3—H3	0.9500	C51—H51A	0.9900
C4—C5	1.386 (4)	C51—H51B	0.9900
C4—C7	1.515 (3)	C52—C53	1.469 (5)
C5—C6	1.399 (4)	C52—H52A	0.9900
C6—H6	0.9500	C52—H52B	0.9900
C7—C8	1.523 (4)	C53—N2	1.165 (6)
C7—H7A	0.9900	N1B—C48B	1.161 (7)
C7—H7B	0.9900	C48B—C49B	1.472 (6)
C8—C13	1.383 (3)	C49B—C50B	1.460 (9)
C8—C9	1.395 (4)	C49B—H49C	0.9900
C9—C10	1.388 (4)	C49B—H49D	0.9900
C10—C11	1.391 (4)	C50B—C51B	1.572 (10)
C10—H10	0.9500	C50B—H50C	0.9900
C11—C12	1.387 (4)	C50B—H50D	0.9900
C11—C14	1.516 (4)	C51B—C52B	1.458 (10)
C12—C13	1.380 (4)	C51B—H51C	0.9900
C13—H13	0.9500	C51B—H51D	0.9900
C14—C15	1.520 (4)	C52B—C53B	1.478 (6)
C14—H14A	0.9900	C52B—H52C	0.9900
C14—H14B	0.9900	C52B—H52D	0.9900
C15—C20	1.384 (4)	C53B—N2B	1.165 (7)
C15—C16	1.391 (4)		
			
C2—O1A—C36A	119.1 (3)	O6—C19—C18	116.4 (2)
O1A—C36A—C37A	108.3 (3)	C20—C19—C18	120.0 (3)
O1A—C36A—H36A	110.0	C15—C20—C19	121.7 (3)
C37A—C36A—H36A	110.0	C15—C20—H20	119.1
O1A—C36A—H36B	110.0	C19—C20—H20	119.1
C37A—C36A—H36B	110.0	C22—C21—C18	110.6 (2)
H36A—C36A—H36B	108.4	C22—C21—H21A	109.5
C38A—C37A—C36A	113.5 (4)	C18—C21—H21A	109.5
C38A—C37A—H37A	108.9	C22—C21—H21B	109.5
C36A—C37A—H37A	108.9	C18—C21—H21B	109.5
C38A—C37A—H37B	108.9	H21A—C21—H21B	108.1
C36A—C37A—H37B	108.9	C27—C22—C23	118.6 (3)
H37A—C37A—H37B	107.7	C27—C22—C21	121.0 (3)
C37A—C38A—C39A	112.7 (4)	C23—C22—C21	120.4 (3)
C37A—C38A—H38A	109.1	O7—C23—C24	123.8 (3)
C39A—C38A—H38A	109.1	O7—C23—C22	116.0 (3)
C37A—C38A—H38B	109.1	C24—C23—C22	120.2 (3)
C39A—C38A—H38B	109.1	C25—C24—C23	121.2 (3)
H38A—C38A—H38B	107.8	C25—C24—H24	119.4
C38A—C39A—Br1A	110.0 (3)	C23—C24—H24	119.4
C38A—C39A—H39C	109.5	C24—C25—C26	119.0 (2)
Br1A—C39A—H39C	0.9	C24—C25—C28	120.2 (2)
C38A—C39A—H39D	109.5	C26—C25—C28	120.8 (2)
Br1A—C39A—H39D	108.6	O8—C26—C25	116.9 (2)
H39C—C39A—H39D	109.5	O8—C26—C27	123.6 (3)
C38A—C39A—H39E	109.5	C25—C26—C27	119.5 (3)
Br1A—C39A—H39E	109.8	C22—C27—C26	121.5 (3)
H39C—C39A—H39E	109.5	C22—C27—H27	119.3
H39D—C39A—H39E	109.5	C26—C27—H27	119.3
C38A—C39A—H39A	109.7	C25—C28—C29	112.13 (19)
Br1A—C39A—H39A	109.7	C25—C28—H28A	109.2
H39C—C39A—H39A	109.3	C29—C28—H28A	109.2
H39D—C39A—H39A	109.4	C25—C28—H28B	109.2
H39E—C39A—H39A	0.2	C29—C28—H28B	109.2
C38A—C39A—H39B	109.7	H28A—C28—H28B	107.9
Br1A—C39A—H39B	109.7	C34—C29—C30	118.0 (2)
H39C—C39A—H39B	110.6	C34—C29—C28	120.2 (2)
H39D—C39A—H39B	1.4	C30—C29—C28	121.8 (2)
H39E—C39A—H39B	108.2	O9—C30—C29	116.1 (2)
H39A—C39A—H39B	108.2	O9—C30—C31	123.2 (2)
O1B—C2—C1	119.4 (5)	C29—C30—C31	120.7 (2)
O1A—C2—C1	116.4 (2)	C30—C31—C32	121.0 (2)
O1C—C2—C1	118.5 (5)	C30—C31—H31	119.5
O1B—C2—C3	119.9 (5)	C32—C31—H31	119.5
O1A—C2—C3	122.8 (3)	C33—C32—C31	118.4 (2)
O1C—C2—C3	119.2 (5)	C33—C32—C35	122.2 (2)
C1—C2—C3	120.8 (2)	C31—C32—C35	119.4 (2)
C2—O1B—C36B	116 (3)	O10—C33—C32	116.5 (2)
O1B—C36B—C37B	108.6 (17)	O10—C33—C34	123.2 (2)
O1B—C36B—H36C	110.0	C32—C33—C34	120.4 (2)
C37B—C36B—H36C	110.0	C33—C34—C29	121.6 (2)
O1B—C36B—H36D	110.0	C33—C34—H34	119.2
C37B—C36B—H36D	110.0	C29—C34—H34	119.2
H36C—C36B—H36D	108.3	C1—C35—C32	111.1 (2)
C38B—C37B—C36B	111.8 (8)	C1—C35—H35A	109.4
C38B—C37B—H37C	109.3	C32—C35—H35A	109.4
C36B—C37B—H37C	109.3	C1—C35—H35B	109.4
C38B—C37B—H37D	109.3	C32—C35—H35B	109.4
C36B—C37B—H37D	109.3	H35A—C35—H35B	108.0
H37C—C37B—H37D	107.9	O3—C40—H40A	109.5
C37B—C38B—C39B	111.9 (8)	O3—C40—H40B	109.5
C37B—C38B—H38C	109.2	H40A—C40—H40B	109.5
C39B—C38B—H38C	109.2	O3—C40—H40C	109.5
C37B—C38B—H38D	109.2	H40A—C40—H40C	109.5
C39B—C38B—H38D	109.2	H40B—C40—H40C	109.5
H38C—C38B—H38D	107.9	O4—C41—H41A	109.5
C38B—C39B—Br1B	124.6 (15)	O4—C41—H41B	109.5
C38B—C39B—H39F	106.2	H41A—C41—H41B	109.5
Br1B—C39B—H39F	106.2	O4—C41—H41C	109.5
C38B—C39B—H39G	106.2	H41A—C41—H41C	109.5
Br1B—C39B—H39G	106.2	H41B—C41—H41C	109.5
H39F—C39B—H39G	106.4	O4—C41A—H41D	109.5
C2—O1C—C36C	119.6 (10)	O4—C41A—H41E	109.5
O1C—C36C—C37C	107.4 (13)	H41D—C41A—H41E	109.5
O1C—C36C—H36G	110.2	O4—C41A—H41F	109.5
C37C—C36C—H36G	110.2	H41D—C41A—H41F	109.5
O1C—C36C—H36H	110.2	H41E—C41A—H41F	109.5
C37C—C36C—H36H	110.2	O5—C42—H42A	109.5
H36G—C36C—H36H	108.5	O5—C42—H42B	109.5
C38C—C37C—C36C	119.3 (15)	H42A—C42—H42B	109.5
C38C—C37C—H37E	107.5	O5—C42—H42C	109.5
C36C—C37C—H37E	107.5	H42A—C42—H42C	109.5
C38C—C37C—H37F	107.5	H42B—C42—H42C	109.5
C36C—C37C—H37F	107.5	O6—C43—H43A	109.5
H37E—C37C—H37F	107.0	O6—C43—H43B	109.5
C37C—C38C—C39C	121.1 (15)	H43A—C43—H43B	109.5
C37C—C38C—H38E	107.1	O6—C43—H43C	109.5
C39C—C38C—H38E	107.1	H43A—C43—H43C	109.5
C37C—C38C—H38F	107.1	H43B—C43—H43C	109.5
C39C—C38C—H38F	107.1	O7—C44—H44A	109.5
H38E—C38C—H38F	106.8	O7—C44—H44B	109.5
C38C—C39C—Br1C	118.4 (14)	H44A—C44—H44B	109.5
C38C—C39C—H39H	107.7	O7—C44—H44C	109.5
Br1C—C39C—H39H	107.7	H44A—C44—H44C	109.5
C38C—C39C—H39I	107.7	H44B—C44—H44C	109.5
Br1C—C39C—H39I	107.7	O8—C45—H45A	109.5
H39H—C39C—H39I	107.1	O8—C45—H45B	109.5
C5—O2—H2	110 (3)	H45A—C45—H45B	109.5
C9—O3—C40	117.9 (3)	O8—C45—H45C	109.5
C41A—O4—C12	118.7 (13)	H45A—C45—H45C	109.5
C12—O4—C41	116.1 (2)	H45B—C45—H45C	109.5
C16—O5—C42	118.4 (3)	O9—C46—H46A	109.5
C19—O6—C43	118.5 (2)	O9—C46—H46B	109.5
C23—O7—C44	117.8 (3)	H46A—C46—H46B	109.5
C26—O8—C45	118.5 (2)	O9—C46—H46C	109.5
C30—O9—C46	118.4 (2)	H46A—C46—H46C	109.5
C33—O10—C47	118.4 (2)	H46B—C46—H46C	109.5
C6—C1—C2	117.8 (2)	O10—C47—H47A	109.5
C6—C1—C35	120.7 (2)	O10—C47—H47B	109.5
C2—C1—C35	121.4 (2)	H47A—C47—H47B	109.5
C2—C3—C4	121.2 (2)	O10—C47—H47C	109.5
C2—C3—H3	119.4	H47A—C47—H47C	109.5
C4—C3—H3	119.4	H47B—C47—H47C	109.5
C5—C4—C3	117.7 (2)	N1—C48—C49	174.1 (15)
C5—C4—C7	121.5 (2)	C48—C49—C50	112.6 (7)
C3—C4—C7	120.8 (2)	C48—C49—H49A	109.1
O2—C5—C4	118.1 (2)	C50—C49—H49A	109.1
O2—C5—C6	121.2 (2)	C48—C49—H49B	109.1
C4—C5—C6	120.7 (2)	C50—C49—H49B	109.1
C1—C6—C5	121.8 (2)	H49A—C49—H49B	107.8
C1—C6—H6	119.1	C49—C50—C51	106.9 (6)
C5—C6—H6	119.1	C49—C50—H50A	110.3
C4—C7—C8	112.4 (2)	C51—C50—H50A	110.3
C4—C7—H7A	109.1	C49—C50—H50B	110.3
C8—C7—H7A	109.1	C51—C50—H50B	110.3
C4—C7—H7B	109.1	H50A—C50—H50B	108.6
C8—C7—H7B	109.1	C52—C51—C50	108.3 (6)
H7A—C7—H7B	107.8	C52—C51—H51A	110.0
C13—C8—C9	117.4 (2)	C50—C51—H51A	110.0
C13—C8—C7	121.0 (2)	C52—C51—H51B	110.0
C9—C8—C7	121.6 (2)	C50—C51—H51B	110.0
O3—C9—C10	123.8 (2)	H51A—C51—H51B	108.4
O3—C9—C8	115.4 (2)	C53—C52—C51	111.5 (7)
C10—C9—C8	120.8 (2)	C53—C52—H52A	109.3
C9—C10—C11	121.0 (3)	C51—C52—H52A	109.3
C9—C10—H10	119.5	C53—C52—H52B	109.3
C11—C10—H10	119.5	C51—C52—H52B	109.3
C12—C11—C10	118.0 (2)	H52A—C52—H52B	108.0
C12—C11—C14	121.4 (2)	N2—C53—C52	174.5 (14)
C10—C11—C14	120.5 (3)	N1B—C48B—C49B	168 (3)
C13—C12—C11	120.6 (2)	C50B—C49B—C48B	112.7 (12)
C13—C12—O4	120.2 (3)	C50B—C49B—H49C	109.0
C11—C12—O4	119.1 (2)	C48B—C49B—H49C	109.0
C12—C13—C8	122.0 (3)	C50B—C49B—H49D	109.0
C12—C13—H13	119.0	C48B—C49B—H49D	109.0
C8—C13—H13	119.0	H49C—C49B—H49D	107.8
C11—C14—C15	113.0 (2)	C49B—C50B—C51B	107.2 (9)
C11—C14—H14A	109.0	C49B—C50B—H50C	110.3
C15—C14—H14A	109.0	C51B—C50B—H50C	110.3
C11—C14—H14B	109.0	C49B—C50B—H50D	110.3
C15—C14—H14B	109.0	C51B—C50B—H50D	110.3
H14A—C14—H14B	107.8	H50C—C50B—H50D	108.5
C20—C15—C16	118.5 (2)	C52B—C51B—C50B	108.5 (10)
C20—C15—C14	120.2 (3)	C52B—C51B—H51C	110.0
C16—C15—C14	121.2 (3)	C50B—C51B—H51C	110.0
O5—C16—C15	116.2 (2)	C52B—C51B—H51D	110.0
O5—C16—C17	124.1 (3)	C50B—C51B—H51D	110.0
C15—C16—C17	119.6 (3)	H51C—C51B—H51D	108.4
C18—C17—C16	121.5 (3)	C51B—C52B—C53B	111.0 (10)
C18—C17—H17	119.3	C51B—C52B—H52C	109.4
C16—C17—H17	119.3	C53B—C52B—H52C	109.4
C17—C18—C19	118.6 (3)	C51B—C52B—H52D	109.4
C17—C18—C21	120.2 (3)	C53B—C52B—H52D	109.4
C19—C18—C21	121.1 (3)	H52C—C52B—H52D	108.0
O6—C19—C20	123.6 (3)	N2B—C53B—C52B	171 (2)
			
C2—O1A—C36A—C37A	173.6 (4)	C14—C15—C16—C17	−177.5 (2)
O1A—C36A—C37A—C38A	77.7 (5)	O5—C16—C17—C18	−178.4 (3)
C36A—C37A—C38A—C39A	178.6 (3)	C15—C16—C17—C18	1.6 (4)
C37A—C38A—C39A—Br1A	−179.2 (3)	C16—C17—C18—C19	−2.0 (4)
C36A—O1A—C2—C1	−156.6 (4)	C16—C17—C18—C21	176.4 (3)
C36A—O1A—C2—C3	21.6 (7)	C43—O6—C19—C20	−0.5 (4)
C1—C2—O1B—C36B	118 (3)	C43—O6—C19—C18	179.7 (3)
C3—C2—O1B—C36B	−62 (4)	C17—C18—C19—O6	−179.3 (2)
C2—O1B—C36B—C37B	−172 (3)	C21—C18—C19—O6	2.3 (4)
O1B—C36B—C37B—C38B	157 (4)	C17—C18—C19—C20	0.9 (4)
C36B—C37B—C38B—C39B	170 (4)	C21—C18—C19—C20	−177.5 (2)
C37B—C38B—C39B—Br1B	18 (7)	C16—C15—C20—C19	−1.2 (4)
C1—C2—O1C—C36C	159 (2)	C14—C15—C20—C19	176.4 (2)
C3—C2—O1C—C36C	−7 (4)	O6—C19—C20—C15	−179.1 (2)
C2—O1C—C36C—C37C	177 (3)	C18—C19—C20—C15	0.7 (4)
O1C—C36C—C37C—C38C	144 (3)	C17—C18—C21—C22	−95.5 (3)
C36C—C37C—C38C—C39C	169 (2)	C19—C18—C21—C22	82.9 (4)
C37C—C38C—C39C—Br1C	48 (3)	C18—C21—C22—C27	−92.7 (3)
O1B—C2—C1—C6	−178 (2)	C18—C21—C22—C23	85.5 (3)
O1A—C2—C1—C6	179.6 (4)	C44—O7—C23—C24	2.5 (4)
O1C—C2—C1—C6	−164.5 (19)	C44—O7—C23—C22	−178.1 (3)
C3—C2—C1—C6	1.4 (4)	C27—C22—C23—O7	179.7 (2)
O1B—C2—C1—C35	5 (2)	C21—C22—C23—O7	1.4 (4)
O1A—C2—C1—C35	2.4 (5)	C27—C22—C23—C24	−1.0 (4)
O1C—C2—C1—C35	18.3 (19)	C21—C22—C23—C24	−179.2 (2)
C3—C2—C1—C35	−175.8 (2)	O7—C23—C24—C25	−178.1 (2)
O1B—C2—C3—C4	180 (2)	C22—C23—C24—C25	2.6 (4)
O1A—C2—C3—C4	−178.0 (4)	C23—C24—C25—C26	−1.6 (4)
O1C—C2—C3—C4	165.9 (19)	C23—C24—C25—C28	177.4 (2)
C1—C2—C3—C4	0.1 (4)	C45—O8—C26—C25	−179.1 (2)
C2—C3—C4—C5	−1.0 (4)	C45—O8—C26—C27	0.1 (4)
C2—C3—C4—C7	177.9 (2)	C24—C25—C26—O8	178.4 (2)
C3—C4—C5—O2	−179.6 (2)	C28—C25—C26—O8	−0.7 (3)
C7—C4—C5—O2	1.6 (4)	C24—C25—C26—C27	−0.9 (4)
C3—C4—C5—C6	0.4 (4)	C28—C25—C26—C27	−179.9 (2)
C7—C4—C5—C6	−178.5 (2)	C23—C22—C27—C26	−1.6 (4)
C2—C1—C6—C5	−2.0 (4)	C21—C22—C27—C26	176.6 (2)
C35—C1—C6—C5	175.2 (2)	O8—C26—C27—C22	−176.7 (2)
O2—C5—C6—C1	−178.9 (2)	C25—C26—C27—C22	2.5 (4)
C4—C5—C6—C1	1.1 (4)	C24—C25—C28—C29	−86.0 (3)
C5—C4—C7—C8	90.5 (3)	C26—C25—C28—C29	93.1 (3)
C3—C4—C7—C8	−88.3 (3)	C25—C28—C29—C34	−96.5 (3)
C4—C7—C8—C13	−88.3 (3)	C25—C28—C29—C30	83.0 (3)
C4—C7—C8—C9	89.8 (3)	C46—O9—C30—C29	179.1 (3)
C40—O3—C9—C10	−1.1 (4)	C46—O9—C30—C31	−0.8 (4)
C40—O3—C9—C8	178.9 (3)	C34—C29—C30—O9	−179.4 (2)
C13—C8—C9—O3	−179.4 (2)	C28—C29—C30—O9	1.1 (3)
C7—C8—C9—O3	2.4 (3)	C34—C29—C30—C31	0.6 (3)
C13—C8—C9—C10	0.6 (3)	C28—C29—C30—C31	−178.9 (2)
C7—C8—C9—C10	−177.6 (2)	O9—C30—C31—C32	179.5 (2)
O3—C9—C10—C11	179.9 (2)	C29—C30—C31—C32	−0.5 (4)
C8—C9—C10—C11	−0.2 (4)	C30—C31—C32—C33	−0.3 (3)
C9—C10—C11—C12	−0.3 (4)	C30—C31—C32—C35	178.4 (2)
C9—C10—C11—C14	178.4 (2)	C47—O10—C33—C32	176.2 (3)
C10—C11—C12—C13	0.3 (3)	C47—O10—C33—C34	−4.6 (4)
C14—C11—C12—C13	−178.4 (2)	C31—C32—C33—O10	−179.8 (2)
C10—C11—C12—O4	177.9 (2)	C35—C32—C33—O10	1.5 (3)
C14—C11—C12—O4	−0.8 (3)	C31—C32—C33—C34	1.0 (4)
C41A—O4—C12—C13	−2 (2)	C35—C32—C33—C34	−177.7 (2)
C41—O4—C12—C13	−72.5 (4)	O10—C33—C34—C29	180.0 (2)
C41A—O4—C12—C11	−180 (2)	C32—C33—C34—C29	−0.9 (4)
C41—O4—C12—C11	109.8 (3)	C30—C29—C34—C33	0.1 (4)
C11—C12—C13—C8	0.2 (4)	C28—C29—C34—C33	179.6 (2)
O4—C12—C13—C8	−177.4 (2)	C6—C1—C35—C32	−85.2 (3)
C9—C8—C13—C12	−0.7 (3)	C2—C1—C35—C32	91.9 (3)
C7—C8—C13—C12	177.6 (2)	C33—C32—C35—C1	94.0 (3)
C12—C11—C14—C15	84.9 (3)	C31—C32—C35—C1	−84.6 (3)
C10—C11—C14—C15	−93.7 (3)	C48—C49—C50—C51	−178.9 (10)
C11—C14—C15—C20	−81.1 (3)	C49—C50—C51—C52	179.2 (8)
C11—C14—C15—C16	96.4 (3)	C50—C51—C52—C53	173.2 (9)
C42—O5—C16—C15	177.3 (3)	N1B—C48B—C49B—C50B	88 (16)
C42—O5—C16—C17	−2.6 (4)	C48B—C49B—C50B—C51B	177.6 (17)
C20—C15—C16—O5	−179.9 (2)	C49B—C50B—C51B—C52B	−173.5 (14)
C14—C15—C16—O5	2.6 (4)	C50B—C51B—C52B—C53B	−170.6 (16)
C20—C15—C16—C17	0.0 (4)		

### A1/A2-Bromobutoxy–hydroxy difunctionalized pillar[5]arene– hexanedinitrile (1/1) Hydrogen-bond geometry (Å, º)

**Table:**

*D*—H···*A*	*D*—H	H···*A*	*D*···*A*	*D*—H···*A*
C37*A*—H37*A*···O6^i^	0.99	2.60	3.587 (5)	172
O2—H2···N2^ii^	0.89 (4)	1.98 (4)	2.866 (10)	178 (4)
O2—H2···N2*B*^ii^	0.89 (4)	2.11 (4)	2.980 (14)	168 (4)
C20—H20···O4	0.95	2.51	3.171 (3)	127
C27—H27···O6	0.95	2.66	3.186 (3)	116
C40—H40*B*···N1	0.98	2.67	3.535 (16)	148

Symmetry codes: (i) −*x*+3/2, *y*−1/2, −*z*+3/2; (ii) −*x*+1, −*y*+1, −*z*+2.

## References

[bb1] Al-Azemi, T. F. & Vinodh, M. (2020). *Polym. Chem.* **11**, 3305–3312.

[bb2] Al-Azemi, T. F. & Vinodh, M. (2021). *RSC Adv.* **11**, 2995–3002. 10.1039/d1ra00078k PMC8693802 35424224

[bb3] Al-Azemi, T. F. & Vinodh, M. (2022). *RSC Adv.* **12**, 1797–1806. 10.1039/d1ra09043g PMC8979204 35425178

[bb4] Bojtár, M., Simon, A., Bombicz, P. & Bitter, I. (2017). *Org. Lett.* **19**, 4528–4531. 10.1021/acs.orglett.7b02092 28825839

[bb5] Ding, J., Chen, J., Mao, W., Huang, J. & Ma, D. (2017). *Org. Biomol. Chem.* **15**, 7894–7897. 10.1039/c7ob02013a 28891584

[bb6] Fang, Y., Deng, Y. & Dehaen, W. (2020). *Coord. Chem. Rev.* **415**, 213313.

[bb7] Groom, C. R., Bruno, I. J., Lightfoot, M. P. & Ward, S. C. (2016). *Acta Cryst.* B**72**, 171–179. 10.1107/S2052520616003954 PMC4822653 27048719

[bb8] Guo, F., Sun, Y., Xi, B. & Diao, G. (2018). *Supramol. Chem.* **30**, 81–92.

[bb9] Guo, L., Du, J., Wang, Y., Shi, K. & Ma, E. (2020). *J. Incl Phenom. Macrocycl. Chem.* **97**, 1–17.

[bb10] Higashi, T. (1995). *ABSCOR*. Rigaku Corporation, Tokyo, Japan. Huo, G.-F., Han, Y., Sun, J. & Yan, C.-G. (2016). *J. Incl. Phenom. Macrocycl. Chem.* **86**, 231–240.

[bb11] Hu, W.-B., Xie, C.-D., Hu, W.-J., Zhao, X.-L., Liu, Y. A., Huo, J.-C., Li, J. S., Jiang, B. & Wen, K. (2015). *J. Org. Chem.* **80**, 7994–8000. 10.1021/acs.joc.5b01038 26219027

[bb12] Ji, J., Li, Y., Xiao, C., Cheng, G., Luo, K., Gong, Q., Zhou, D., Chruma, J. J., Wu, W. & Yang, C. (2020). *Chem. Commun.* **56**, 161–164. 10.1039/c9cc08541f 31799971

[bb13] Kim, S., Park, I.-H., Lee, E., Jung, J. H. & Lee, S. S. (2022). *Inorg. Chem.* **61**, 7069–7074. 10.1021/acs.inorgchem.2c00514 35482519

[bb14] Lee, E., Park, I.-H., Ju, H., Kim, S., Jung, J. H., Habata, Y. & Lee, S. S. (2019*a*). *Angew. Chem. Int. Ed.* **58**, 11296–11300. 10.1002/anie.201904183 31209942

[bb15] Lee, E., Ryu, H., Ju, H., Kim, S., Lee, J.-E., Jung, J. H., Kuwahara, S., Ikeda, M., Habata, Y. & Lee, S. S. (2019*b*). *Chem. Eur. J.* **25**, 949–953. 10.1002/chem.201805275 30450626

[bb16] Li, Q., Zhu, H. & Huang, F. (2020). *Trends Chem.* **2**, 850–864.

[bb17] Macrae, C. F., Sovago, I., Cottrell, S. J., Galek, P. T. A., McCabe, P., Pidcock, E., Platings, M., Shields, G. P., Stevens, J. S., Towler, M. & Wood, P. A. (2020). *J. Appl. Cryst.* **53**, 226–235. 10.1107/S1600576719014092 PMC6998782 32047413

[bb18] McKinnon, J. J., Jayatilaka, D. & Spackman, M. A. (2007). *Chem. Commun.* pp. 3814–3816. 10.1039/b704980c 18217656

[bb19] Ogoshi, T., Yamagishi, T. & Nakamoto, Y. (2016). *Chem. Rev.* **116**, 7937–8002. 10.1021/acs.chemrev.5b00765 27337002

[bb20] Rigaku (2016). *CrystalClear-SM Expert*. Rigaku Corporation, Tokyo, Japan.

[bb21] Rigaku (2017). *CrystalStructure*. Rigaku Corporation, Tokyo, Japan.

[bb22] Sheldrick, G. M. (2015). *Acta Cryst.* A**71**, 3–8.

[bb23] Strutt, N. L., Schneebeli, S. T. & Stoddart, J. F. (2013). *Supramol. Chem.* **25**, 596–608.

[bb24] Turner, M. J., McKinnon, J. J., Wolff, S. K., Grimwood, D. J., Spackman, P. R., Jayatilaka, D. & Spackman, M. A. (2017). *CrystalExplorer 17*. University of Western Australia.

[bb25] Vinodh, M., Alipour, F. H. & Al-Azemi, T. F. (2023). *ACS Omega*, **8**, 1466–1475. 10.1021/acsomega.2c06903 PMC9835175 36643541

[bb26] Wang, K., Tian, X., Jordan, J. H., Velmurugan, K., Wang, L. & Hu, X.-Y. (2022*a*). *Chin. Chem. Lett.* **33**, 2022, 89–96.

[bb27] Wang, Z., Liu, Y. A., Yang, H., Hu, W.-B. & Wen, K. (2022*b*). *Org. Lett.* **24**, 1822–1826. 10.1021/acs.orglett.2c00272 35225626

[bb28] Yang, W., Zhang, W., Chen, J. & Zhou, J. (2024). *Chin. Chem. Lett.* **35**, 108740.

[bb29] Yang, Y.-F., Hu, W.-B., Shi, L., Li, S.-G., Zhao, X.-L., Liu, Y. A., Li, J.-S., Jiang, B. & Wen, K. (2018). *Org. Biomol. Chem.* **16**, 2028–2032. 10.1039/c8ob00156a 29460948

[bb30] Zhang, T., Wang, K., Huang, X., Jiao, J. & Hu, X.-Y. (2023). *Chem. Eur. J.* **29**, e202203738. 10.1002/chem.202203738 36595380

[bb31] Zhu, H., Li, Q., Khalil-Cruz, L. E., Khashab, N. M., Yu, G. & Huang, F. (2021). *Sci. China Chem*. **64**, 688-700.

[bb32] Zyryanov, G. V., Kopchuk, D. S., Kovalev, I. S., Santra, S., Majee, A. & Ranu, B. C. (2023). *Int. J. Mol. Sci.* **24**, 5167. 10.3390/ijms24065167 PMC10049520 36982244

