# Supplementary material for: Crystal structure and supra­molecular features of a host–guest inclusion complex based on A1/A2-hetero-difunctionalized pillar[5]arene
Source: Acta Crystallogr E Crystallogr Commun. 2024 Sep 24;80(Pt 10):1069–74. doi: 10.1107/S2056989024009216 (PMC11451480; doi:10.1107/S2056989024009216)
Supplement: Supplementary file 4 [file e-80-01069-sup5.doc]

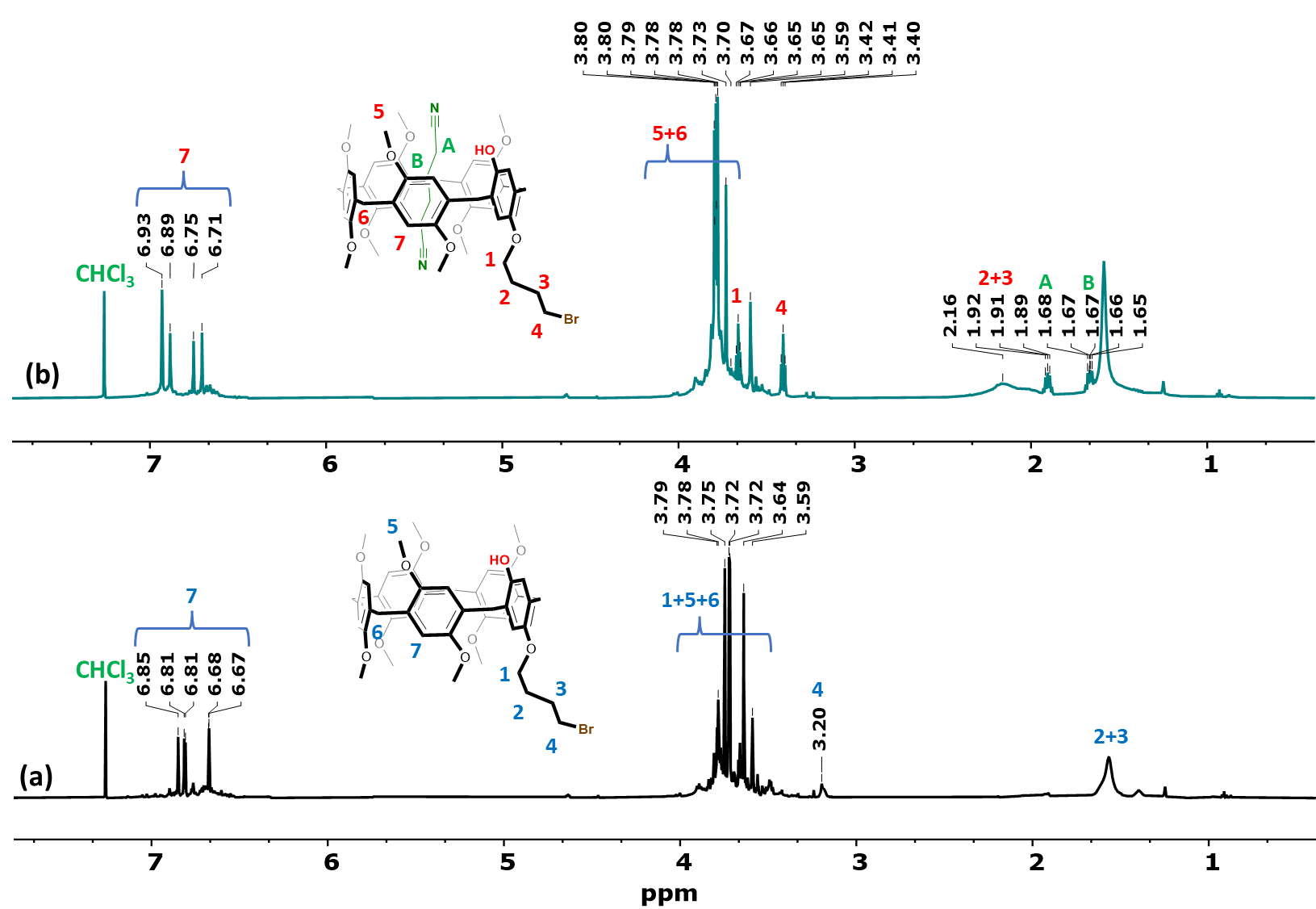


**Figure S1.** ^1^HNMR (600 MHz, CDCl_3_ at 298 K) spectra of A1/A2-bromobutoxy-hydroxy difunctionalized pillar[5]arene (**PilButBrOH**) before (**a**), and after the addition of one equivalent of the adiponitrile guest (**b**).


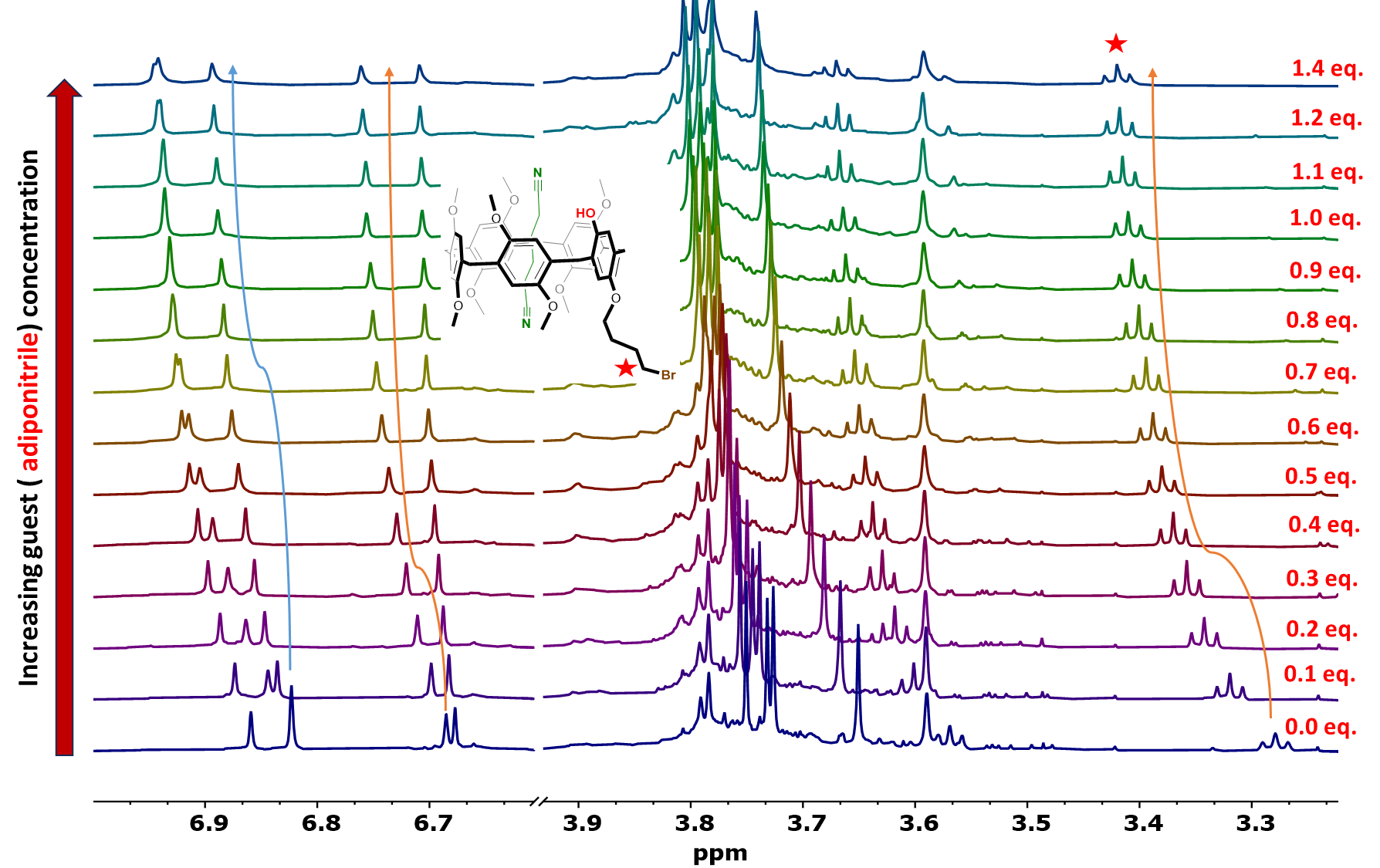


**Figure S2.** Expanded ^1^HNMR (600 MHz, CDCl_3_ at 298 K) titration spectra of A1/A2-bromobutoxy-hydroxy difunctionalized pillar[5]arene (**PilButBrOH)** after sequential additions of adiponitrile guest, **ADN** (0 → 1.4 equivalents).
